# Insights Into Chloroplast Genome Evolution Across Opuntioideae (Cactaceae) Reveals Robust Yet Sometimes Conflicting Phylogenetic Topologies

**DOI:** 10.3389/fpls.2020.00729

**Published:** 2020-06-19

**Authors:** Matias Köhler, Marcelo Reginato, Tatiana Teixeira Souza-Chies, Lucas C. Majure

**Affiliations:** ^1^Programa de Pós-Graduação em Botânica, Universidade Federal do Rio Grande do Sul, Porto Alegre, Brazil; ^2^Florida Museum of Natural History, University of Florida Herbarium (FLAS), Gainesville, FL, United States; ^3^Department of Research, Conservation and Collections, Desert Botanical Garden, Phoenix, AZ, United States

**Keywords:** cacti, *de novo* assembly, *Opuntia*, plastid structural rearrangements, plastome, pseudogenization, reference-guided assembly

## Abstract

Chloroplast genomes (plastomes) are frequently treated as highly conserved among land plants. However, many lineages of vascular plants have experienced extensive structural rearrangements, including inversions and modifications to the size and content of genes. Cacti are one of these lineages, containing the smallest plastome known for an obligately photosynthetic angiosperm, including the loss of one copy of the inverted repeat (∼25 kb) and the *ndh* gene suite, but only a few cacti from the subfamily Cactoideae have been sufficiently characterized. Here, we investigated the variation of plastome sequences across the second-major lineage of the Cactaceae, the subfamily Opuntioideae, to address (1) how variable is the content and arrangement of chloroplast genome sequences across the subfamily, and (2) how phylogenetically informative are the plastome sequences for resolving major relationships among the clades of Opuntioideae. Our *de novo* assembly of the *Opuntia quimilo* plastome recovered an organelle of 150,347 bp in length with both copies of the inverted repeat and the presence of all the *ndh* gene suite. An expansion of the large single copy unit and a reduction of the small single copy unit was observed, including translocations and inversion of genes, as well as the putative pseudogenization of some loci. Comparative analyses among all clades within Opuntioideae suggested that plastome structure and content vary across taxa of this subfamily, with putative independent losses of the *ndh* gene suite and pseudogenization of genes across disparate lineages, further demonstrating the dynamic nature of plastomes in Cactaceae. Our plastome dataset was robust in resolving three tribes with high support within Opuntioideae: Cylindropuntieae, Tephrocacteae and Opuntieae. However, conflicting topologies were recovered among major clades when exploring different assemblies of markers. A plastome-wide survey for highly informative phylogenetic markers revealed previously unused regions for future use in Sanger-based studies, presenting a valuable dataset with primers designed for continued evolutionary studies across Cactaceae. These results bring new insights into the evolution of plastomes in cacti, suggesting that further analyses should be carried out to address how ecological drivers, physiological constraints and morphological traits of cacti may be related with the common rearrangements in plastomes that have been reported across the family.

## Introduction

Cacti comprise one of the most charismatic plant clades of the world, exhibiting an enormous variety of growth forms, morphology and intriguing niche occupancy across the Americas (Britton and Rose, 1919; Anderson, 2001; Hunt et al., 2006; Hernández-Hernández et al., 2011). This diversity is reflected in a high number of species and heterogeneous diversification rates across the clade (Arakaki et al., 2011; Hernández-Hernández et al., 2014). Some uncommon features in most angiosperms, such as succulent tissues, Crassulacean acid metabolism (CAM), betalain pigments and the reduction of or absence of leaves are typical characters of cacti that have long captured the attention of plant biologists and have been suggested as adaptations to allow survival in harsh environments (Mooney et al., 1977; Mauseth, 1999; Landrum, 2002; Nobel, 2002). Indeed, members of the family are conspicuous elements of the arid and semiarid succulent biome of the New World, but they are also found in subtropical and tropical forests, especially as epiphytes (Taylor and Zappi, 2004; Hunt et al., 2006). Besides major morphological and physiological adaptations, genetic and genomic-level changes are also expected. For example, whole genome duplication events have long been suggested to be associated with adaptations to extreme environments (e.g., Stebbins, 1971; Soltis and Soltis, 2000; Brochmann et al., 2004), and significant gene family expansion in genes related to stress adaptation, as well as more restricted events of gene duplications were reported in lineages of Caryophyllales adapted to severe environments including in cacti (Wang et al., 2019).

Although gene content, structural organization and size of the chloroplast genome (plastomes) of land plants is often considered highly conserved (Raubeson and Jansen, 2005; Chumley et al., 2006; Wicke et al., 2011), deviations have been increasingly reported in some clades and have challenged the generality of this phenomenon (Daniell et al., 2016; Mower and Vickrey, 2018; Ruhlman and Jansen, 2018). Astonishing variety of size have been observed across land plants, from 19 kb in a non-photosynthetic *Epipogium roseum* (D. Don) Lindl. (Orchidaceae) to giant plastomes with 217 kb, as in *Pelargonium* × *hortorum* L. H. Bailey (Geraniaceae) (Chumley et al., 2006; Schelkunov et al., 2015), reflected by expansions or contraction of the inverted repeat (IR), large single copy (LSC) or even small single copy (SSC) units. Also, the independent losses of one copy of the inverted repeat region (∼25 kb in size) have been identified across disparate clades, such as Fabaceae, Geraniaceae, Orobanchaceae, and Cactaceae (Cai et al., 2008; Ruhlman and Jansen, 2014; Sanderson et al., 2015), and a variety of taxa have lost particular genes (e.g., *ndh* genes in parasites, carnivorous plants and xerophytes) (Braukmann et al., 2009; Wicke et al., 2011; Iles et al., 2013; Peredo et al., 2013; Ruhlman et al., 2015; Sanderson et al., 2015).

Members of Cactaceae also have experienced different alterations in their chloroplast genome. A conserved inversion of ∼6 kb on the large single copy unit comprising the *trnM-rbcL* genes have long been suggested (Wallace, 1995) and more recently confirmed (Sanderson et al., 2015; Majure et al., 2019; Solórzano et al., 2019). Besides that, the first cactus plastome assembled from the saguaro cactus [*Carnegiea gigantea* (Engelm.) Britton & Rose] exhibited an exceptional reduction in size (113 kb) and gene content, including the loss of one of the two inverted repeat regions and nine of the 11 *ndh* genes (Sanderson et al., 2015). More recently, newly assembled plastomes of seven species of the short-globose cacti of *Mammillaria* Haw. revealed three different plastome structures across the genus, all with two copies of a divergent inverted repeat, including (i) an extreme reduction in size of IRs [<1 kb, typically ranging from 15 to 30 kb in land plants (Zhu et al., 2016)]; (ii) an intermediate reduction of IR (∼7 kb) with translocation of some typical LSC genes to the IR; and (iii) a structure with a divergent IR structure and a surprisingly reduced plastome (∼107 kb), being now the putative smallest plastome known for an obligately photosynthetic angiosperm (Solórzano et al., 2019). However, considering these dissimilar patterns between the few described plastomes of cacti, a broad sampling including other lineages may shed new insights into chloroplast genome evolution across the family.

The classification of Cactaceae has been long proposed based on morphological characters (Schumann, 1899; Britton and Rose, 1919; Backeberg, 1958; Hunt et al., 2006), and further tested with the aid of molecular phylogenies (Nyffeler, 2002; Bárcenas et al., 2011; Hernández-Hernández et al., 2011). Three major well-supported clades are currently circumscribed as subfamilies: Opuntioideae, Maihuenioideae and Cactoideae, while the traditional “Pereskioideae” has been revealed as a basal grade including the two leafy lineages of the cacti, which are subsequent sisters to the rest, i.e., *Leuenbergeria* Lodé and *Pereskia* Mill. (Edwards et al., 2005, reviewed in Guerrero et al., 2019). Opuntioideae (∼350 spp.) is the most widespread subfamily with members occurring from southern South America (Argentina) to northern North America (Canada) (Britton and Rose, 1919; Anderson, 2001; Hunt et al., 2006; Ritz et al., 2012; Majure and Puente, 2014; Majure et al., 2019). The group shows interesting morphological synapomorphies, such as the small brushlike, barbed spines (i.e., glochids) and a bony aril surrounding a campylotropous ovule (Stuppy, 2002; Taylor et al., 2002). However, the delimitation of taxa within Opuntioideae is still not settled, and the controversy is observed across different taxonomic levels, from species to tribes (Schumann, 1899; Britton and Rose, 1919; Hunt, 2002; Stuppy, 2002; Taylor et al., 2002). Traditional classifications based on general morphology – such as growth form, stem and leaf morphology, as well as floral, fruit, pollen, and seed characters – were used to divide the subfamily from few to up to 20 smaller genera (Britton and Rose, 1919; Stuppy, 2002; Hunt et al., 2006; Griffith and Porter, 2009). Nonetheless, molecular phylogenetic studies, mainly based on chloroplast (*rpl16* intron and *trnL-trnF* region) and nuclear ribosomal ITS sequences, revealed that the most comprehensive genus, *Opuntia* s.l. (L.) Mill., was paraphyletic, which reinforced the recognition of numerous smaller genera corresponding to well-supported clades (Stuppy, 2002; Taylor et al., 2002; Wallace and Dickie, 2002; Griffith and Porter, 2009; Majure et al., 2012; Ritz et al., 2012; Majure and Puente, 2014). Likewise, the tribal classification of Opuntioideae has been controversial based on different approaches, with up to six tribes proposed (Hunt, 2011). While Doweld (1999) and Wallace and Dickie (2002) proposed five tribes, with different circumscriptions from each other — four were recognized as monophyletic in the last comprehensive molecular study of Opuntioideae (Griffith and Porter, 2009). Despite great improvement in our phylogenetic understanding in Opuntioideae (Griffith and Porter, 2009; Ritz et al., 2012; Majure et al., 2019), support for the relationships among those clades, as well as a better taxon sampling with more molecular markers, still needs to be strengthened.

Apart from the external and internal transcribed spacer (*ETS* and *ITS*) of the nuclear ribosomal repeats (*NRR*) and *ppc* marker, most molecular phylogenies of cacti have been historically based on a few plastid markers (*trnL-trnF*, *rpl16*, *trnK*, and *matK*) (Nyffeler, 2002; Edwards et al., 2005; Korotkova et al., 2010; Arakaki et al., 2011; Bárcenas et al., 2011; Demaio et al., 2011; Hernández-Hernández et al., 2011, 2014; Ritz et al., 2012; Bárcenas, 2016; Vargas-Luna et al., 2018). While these markers have shown to be potentially able to resolve some clades, some relationships are still lacking support (Nyffeler, 2002; Griffith and Porter, 2009; Bárcenas et al., 2011; Hernández-Hernández et al., 2011). In this case, next-generation sequencing (NGS) could be a useful tool, since it has transformed the study of non-model plant taxa in phylogenetic inferences with high throughput data allowing deep resolution across major plant clades (Xi et al., 2012; Ma et al., 2014; Gardner et al., 2016; Zong et al., 2019). NGS data are also showing to be extremely useful for discovering informative regions across genomes, for marker development (Wu et al., 2010; Dong et al., 2012; Ripma et al., 2014; Reginato et al., 2016; Abdullah et al., 2019), as well as to investigate chloroplast genome evolution (Dong et al., 2013; Mower and Vickrey, 2018; Yao et al., 2019). Nevertheless, this approach is still in its infancy across Cactaceae (Majure et al., 2019) and remains a path to be explored.

Here, we investigate the use of next-generation sequencing across Opuntioideae to address two major questions: (1) how homogenous is the content and arrangement of chloroplast genomes across the subfamily? and (2) how phylogenetically informative are chloroplast genome sequences for resolving major relationships among the clades of Opuntioideae? We used a combination of *de novo* and reference-guided assemblies to process genome skimming data: (i) assembling and characterizing the first chloroplast genome of an *Opuntia* species, *O. quimilo* K. Schum., (ii) investigating overall patterns of reference-guided assemblies and comparative chloroplast genome sequence analyses across the subfamily, (iii) inferring phylogenetic relationships with assembled sequences and (iv) surveying plastomes for highly informative phylogenetic markers for Sanger-based studies for future use.

## Materials and Methods

### Taxon Sampling, DNA Extraction, and Sequencing

All currently recognized genera in Opuntioideae (sensu Hunt et al., 2006, plus Majure et al., 2019 for *Grusonia* s.l.), with the exception of *Punotia* (see Ritz et al., 2012), were sampled with one accession per genus, resulting in a dataset of 17 taxa, which were sequenced via genome-skimming (Straub et al., 2012; Majure et al., 2019). All seven genera of tribe Opuntieae were included; five of the six genera in Tephrocacteae were sampled, and all five genera in Cylindropuntieae were included in our analyses. Three additional samples were selected as outgroup taxa [Cactoideae: *Parodia magnifica* (F. Ritter) F. H. Brandt and *Coryphantha macromeris* (Engelm.) Lem.; and *Pereskia*: *Pereskia sacharosa* Griseb.] based on previous studies (Arakaki et al., 2011; Hernández-Hernández et al., 2014). Plant materials were from wild collections or from the Desert Botanical Garden’s living collection (see Supplementary Table S1 for details). DNA was extracted from silica-dried or fresh epidermal tissues using a standard CTAB incubation (Doyle and Doyle, 1987) followed by chloroform/isoamyl alcohol precipitation and silica column-based purification steps, as described in Neubig et al. (2014) and Majure et al. (2019). Whole genomic DNAs were quantified using the Qubit dsDNA BR Assay Kit and Qubit 2.0 Fluorometer (Life Technologies, Carlsbad, CA, United States); high-molecular-weight DNA (>15 kb) samples showing no degradation were considered suitable and sent to Rapid Genomics LLC^1^ (Gainesville, FL, United States) for library preparation and high-throughput sequencing using the Illumina HiSeq X platform with 150 bp paired-end reads. A total of sixty samples were included per lane for sequencing.

### *De novo* Assembly and Data Processing for Chloroplast Genome Sequences

Raw reads were imported into Geneious 11.1.5 (Biomatters, Auckland, New Zealand), and paired reads were set with an expected insert size of 300 bp calculated with BBMap using default setting (Bushnell, 2016). Low quality bases (Q < 20) were trimmed, and all reads shorter than 20 bp were discarded using BBDuk for quality control (Bushnell, 2016). Different methods were employed to assemble the chloroplast genome of the diploid *Opuntia quimilo*. First, a *de novo* assembly was performed with 40% of the reads using the Geneious *de novo* assembler (low/fast sensitivity option, plus default settings). A consensus sequence (with a majority threshold for sequence matching – fewest ambiguities) of each contig greater than 1,000 bp in length was saved. Considering that the Cactaceae plastomes already published have unusual rearrangements, we looked for plastid contigs searching those saved contigs against the *Portulaca oleracea* L. plastome (Portulacaceae, one of the closest relatives of Cactaceae; see Walker et al., 2018) (GenBank accession KY490694, Liu et al., 2017) using MegaBLAST (following parameters proposed from Ripma et al., 2014). Additional chloroplast genome *de novo* assemblies of *O. quimilo* were performed using a set of different pipelines, such as GetOrganelle (Jin et al., 2019) and NOVOPlasty (Dierckxsens et al., 2017) to cross-validate and compare among the assemblies. After checking convergence of the assemblies from the different pipelines and the plastid contig recovered from the Geneious *de novo* assembly, we used the NOVOPlasty circular contig for downstream analyses. Annotations were performed with GeSeq (Tillich et al., 2017), using default parameters to predict protein-coding genes by HMMER profile search and ARAGORN v1.2.38 (Laslett and Canback, 2004); tRNA genes were annotated with tRNAscan-SE v2.0 (Lowe and Chan, 2016), and BLAST searches were used to annotate ribosomal RNA (rRNA), tRNA, and DNA genes conserved in embryophyte plastomes (Wommack et al., 2008). All annotations were cross checked with the “Annotate from” feature in Geneious, transferring annotations with a 50% or greater similarity from the *P. oleracea* plastome, and eventual start/stop codons were manually adjusted with the “Open Read Frame (ORF)” feature from Geneious. The genes that had their structures affected by the insertion of internal stop codons and/or a small ORF, thus did not form their respective full coding sequence (CDS), were annotated as putative pseudogenes. The graphical representation of the *O. quimilo* circular annotated plastome was created in OGDRAW (Lohse et al., 2013; Greiner et al., 2019). To visualize changes in gene order and content, we compared the *O. quimilo* assembly with the canonical gene order of the *P. oleracea* plastome via multiple whole genome alignments using MAUVE (default options, assuming colinearity; Darling et al., 2004). Boundaries between the IRa, IRb, LSC, SSC and putative inversions were visually checked in Geneious using an *in silico* approach adapted from Oliver et al. (2010).

### Comparative Chloroplast Genome Sequence Analyses Across Opuntioideae

The newly annotated plastome of *Opuntia quimilo*, with one of the inverted repeats (IRa) manually stripped to avoid data duplication, was then used for a reference guided assembly on the trimmed reads from all other taxa using Geneious mapper with a medium-low sensitivity iterating up to five times (adapted from Ripma et al., 2014). Each of the assemblies mapped had a majority threshold consensus sequence generated and annotations transferred from the *O. quimilo* reference, and manually adjusted. To identify highly variable regions across the subfamily, the 17 assembled Opuntioideae chloroplast genome sequences were compared using mVista (Frazer et al., 2004) in Shuffle-LAGAN alignment mode (Brudno et al., 2003) using the annotated plastome of *O. quimilo* as a reference. We also used the full chloroplast genome sequence alignment (see below) to calculate nucleotide diversity values (π) to detect highly variable sites among Opuntioideae chloroplast genome sequences. DNA polymorphism analysis was performed on DnaSP v.6.10 (Rozas et al., 2017) using the sliding window analysis with a step size of 200 bp and window length of 800 bp. Assembly maps of raw read coverages from Geneious mapper of each taxon to the *O. quimilo* plastome were also used to visualize and compare the gene content of the chloroplast genome sequences across the subfamily.

### Phylogenetic Analyses and Informative Regions

The assembled chloroplast genome sequences, obtained as described in the previous section, were aligned using MAFFT v. 7 with an automatic strategy search for algorithm selection (Katoh and Standley, 2013), using 200PAM scoring matrix and an open gap penalty of 1.53 (offset value 0.123). The alignment was manually examined for misaligned areas following a similarity criterion (Simmons, 2004). Sequence portions that contained gaps and/or ambiguities across more than 80% of the taxa were stripped using the “Mask Alignments” feature in Geneious. Phylogenetic inference was performed using Maximum Likelihood implemented in RAxML 8.2.4 (Stamatakis, 2014) in the CIPRES Portal (Miller et al., 2010). As RAxML is mainly designed to implement generalized time-reversible molecular models (GTR), we employed the GTR + G model for the entire sequence, which have been suggested for topological reconstruction skipping model selection (Abadi et al., 2019), and GTR + I + G is not recommended by Stamatakis (see RAxML v8.2 manual) given the potential interaction between the I and G parameters. Support values were estimated implementing 1,000 bootstrap pseudoreplicates.

To identify and rank highly phylogenetically informative regions in the Opuntioideae plastomes, we split the full plastome alignment into protein coding sequences (cpCDS – pseudogenes were included here), non-coding sequences (cpNCDS) and intergenic spacers (cpIGS) using the annotated *O. quimilo* plastome. Each individual marker (cpCDS, cpNCDS, cpIGS) was extracted from the above-mentioned alignment, and a Maximum Likelihood tree was inferred with RAxML using GTR + G model (see reasons above) and 100 bootstrap replicates. For each marker, we report the number of variable sites, number of parsimony informative sites (PIS), mean sequence distance (under K80 model), alignment length, mean sequence length, mean bootstrap support and distance to the full chloroplast genome sequence tree (RF distance; Robinson and Foulds, 1981). The metrics were retrieved using functions of the R packages *ape* and *phangorn* (Paradis et al., 2004; Schliep, 2011). Markers were ranked by phylogenetic information using a weighted mean of relative values of the following metrics: number of variable sites (weight = 1), mean bootstrap (weight = 2) and distance to the full plastid tree (weight = 3). We designed primer pairs for the top five markers identified in the previous step with suitable size for PCR amplification (< ∼900 bp). Primers flanking the target regions were designed with Primer3, using the default settings (Rozen and Skaletsky, 2000). All metrics reported, as well as primer design, were considered only for the ingroup (the 17 Opuntioideae chloroplast genome sequences). Further phylogenetic inferences (RAxML, GTR + G, 1000 bootstrap), were performed for a dataset concatenating: (1) the top five markers, (2) the top 10 markers, and (3) the five markers which have primers designed.

## Results

### DNA Sequencing

Runs on Illumina HiSeq X resulted in 227,003,814 reads from 20 samples (17 Opuntioideae and three outgroups), between 5,624,110 and 20,219,350 reads per sample, for a mean read number of 11,350,190 sequences. Reads per sample following quality control were between 5,360,990 and 19,863,298 with a mean post-quality control read pool number of 11,084,834. The GC content of the raw reads ranged from 37.4 to 40.6% with a mean of 38.45% and following quality control were between 36.9 and 40% with a mean of 38%. Detailed results with the number of raw reads, post-quality control and %GC content per taxa are presented in Supplementary Table S1.

### *Opuntia quimilo* Plastome

**FIGURE 1:**
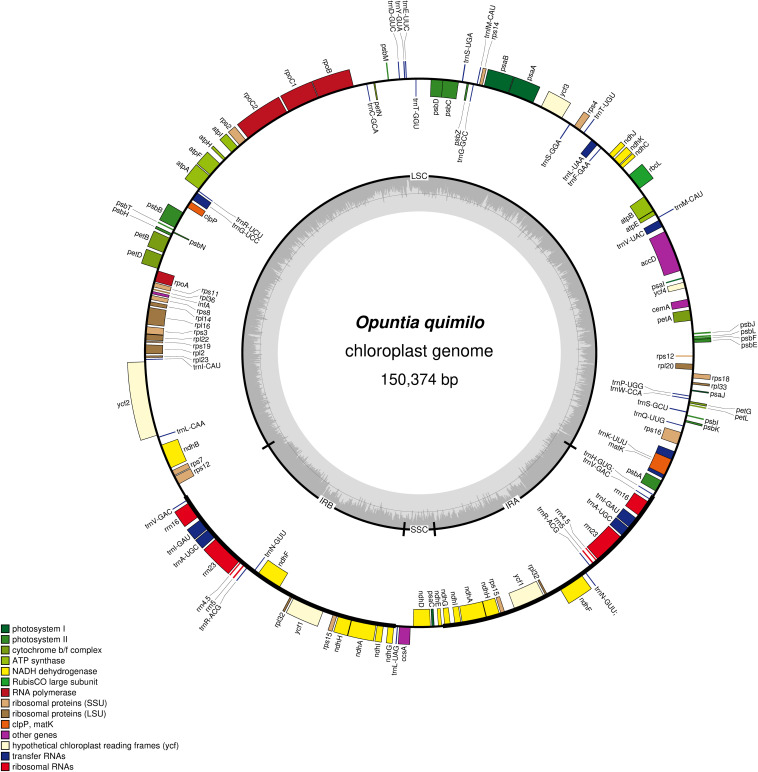
Circular map of chloroplast genome of *Opuntia quimilo* with annotated genes. The genes transcribed clockwise are shown inside of the circle, whereas genes transcribed counter clockwise are shown outside of the circle. The borders of chloroplast genome are defined with LSC, SSC, IRa, and IRb. The dashed gray color of inner circle shows the GC content.

The complete chloroplast genome of *Opuntia quimilo* was sequenced, assembled, annotated and deposited in GenBank (accession number MN114084). The length of the *Opuntia quimilo* plastome is 150,374 bp, including a 101,475 bp LSC region, a 4,115 bp SSC region and 22,392 bp of two IR (IRa and IRb) regions (Figure 1 and Table 1). A total of 701,318 reads were assembled, with an average organelle depth of 844x. The GC content varies from 33% in the SSC, to 35.5% in LSC and 39.6% in the IR regions, while 38% in coding regions (CDS) and 35.6% in non-coding regions (Table 1).

**TABLE 1:** Chloroplast genome composition of *Opuntia quimilo*.

Region	Size (bp)	GC (%)	Genes	CDS	tRNA	rRNA
**Genome**	150.374	36.6	130 (3)	87 (3)	35	8
**LSC**	101.475	35.5	91 (2)	67 (2)	24	0
**SSC**	4.115	33	5	4	1	0
**IRa**	22.392	39.6	17 (1)	8 (1)	5	4
**IRb**	22.392	39.6	17 (1)	8 (1)	5	4

*The number in parentheses indicates pseudogenes (ψ) identified.*

The *de novo* assembly of the Geneious assembler produced 1,000 contigs; of these, 988 were higher than 1,000 bp in length from a minimum length of 1,026 bp to a maximum of 283,150 bp. MegaBLAST search found one consensus plastid contig of 128,909 bp that included the full chloroplast sequence with two putative inverted repeats assembled as a single IR unit (∼22 kb). The GetOrganele and NOVOPlasty pipelines both yielded one plastid contig of 150,374 bp with the same gene content, order, and structure as the plastid contig of the Geneious assembler, except for the two inverted repeats that were interleaved by the LSC and SSC on the first ones, while in Geneious these were merged as one IR.

The *Opuntia quimilo* plastome encodes 87 protein-coding genes (CDS), 35 transfer RNA genes (tRNA) and eight ribosomal RNA (rRNA) genes, totaling 130 genes (Tables 1, 2). Three canonical CDS from angiosperm chloroplast genomes were annotated as putative pseudogenes (Ψ) based on their structure: *accD*, *ycf1*, and *ycf2*. Two of them (*accD* and *ycf2*) are in the LSC, and *ycf1* in the IRs. Duplicated CDS in the IRs included *ndhA*, *ndhF*, *ndhG*, *ndhH*, *ndhI*, *rpl32*, *ycf1*(Ψ), and *rps15*; and all four rRNA genes and five of the 35 tRNAs were duplicated in the IR regions. The *O. quimilo* plastome includes 16 intron-containing genes, of which 15 contain one intron (*atpF*, *ndhA*, *ndhB*, *petB*, *petD, rpl16, rpoC1, rps12*, *rps16, trnA^*UGC*^*, *trnG*^*UCC*^, *trnI*^*GAU*^, *trnK*^*UUU*^, *trnL*^*UAA*^, *trnV*^*UAC*^), while one gene contains two introns (*ycf3*), and the *clpP* gene has lost its two introns, reduced to an exon of 609 bp.

**TABLE 2:** Structural and functional gene composition of *Opuntia quimilo* chloroplast genome.

Gene type	Region	Genes
**(1) Ribosomal RNA (rrn)**	IRa & IRb	*rrn4.5, rrn5, rrn16, rrn23*
**(2) Transfer RNA (trn)**	LSC	trnC^GCA^, trnD^GUC^, trnE^UUC^, trnF^GAA^, trnfM^CAU^, trnG^GCC^, trnG^UCC^*, trnH^GUG^, trnI^CAU^, trnK^UUU^*, trnL^CAA^, trnL^UAA^*, trnM^CAU^, trnP^UGG^, trnQ^UUG^, trnR^UCU^, trnS^GCU^, trnS^GGA^, trnS^UGA^, trnT^GGU^, trnT^UGU^, trnV^UAC^*, trnW^CCA^, trnY^GUA^
	SSC	*trnL*^*UAG*^
	IRa & IRb	trnA^UGC^*, trnI^GAU^*, trnN^GUU^, trnR^ACG^, trnV^GAC^
**(3) Proteins of small subunits of the ribosome (rps)**	LSC	rps2, 3, 4, 7, 8, 11, 12*, 14, 16*, 18, 19
	IRa & IRb	*rps15*
**(4) Proteins of large subunits of the ribosome (rpl)**	LSC	rpl2, 14, 16*, 20, 22, 23, 33, 36
	IRa & IRb	*rpl32*
**(5) DNA dependente RNA polymerase (rpo)**	LSC	rpoA, B, C1*, C2
**(6) NADH dehydrogenase (*ndh*)**	LSC	ndhB*, C, J, K
	SSC	*ndhD, E*
	IRa & IRb	ndhA*, F, G, H, I
**(7) Photosystem I (psa)**	LSC	*psaA, B, I, J*
	SSC	*psaC*
**(8) Photosystem II (psb)**	LSC	*psbA, B, C, D, E, F, H, I, J, K, L, M, N, T, Z*
**(9) Cytochrome b/f complex (pet)**	LSC	petA, B*, D*, G, L, N
**(10) ATP synthase (atp)**	LSC	atpA, B, E, F*, H, I
**(11) Rubisco (rbc)**	LSC	*rbcL*
**(12) Maturase K**	LSC	*matK*
**(13) Protease (clp)**	LSC	*clpP*
**(14) Envelope membrane protein (cem)**	LSC	*cemA*
**(15) Subunit of acetil-CoA-carboxylase (acc)**	LSC	*accD*(Ψ)
**(16) C-type cytochrome synthesis (ccs)**	SSC	*ccsA*
**(17) Translational initiation factor (inf)**	LSC	*infA*
**(18) Hypothetical chloroplast reading frames (ycf)**	LSC	*ycf2*(Ψ), 3**, *4*
	IRa & IRb	*ycf1*(Ψ)

(ψ) Putative pseudogenes. *Gene containing one intron. **Gene containing two introns.

**FIGURE 2:**
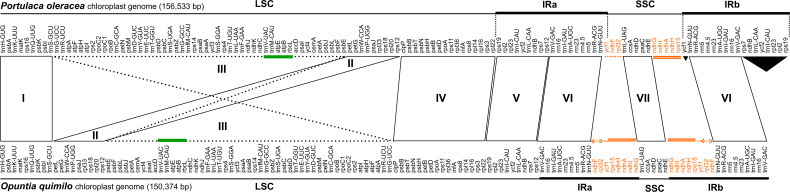
Plastid genome structure and gene order in *Opuntia quimilo* compared with purslane (*Portulaca oleracea*). Purslane has the canonical order typical of most angiosperms. For simplicity, the circular map has been linearized. Green line highlights the *trnM^*CAU*^-rbcL* synapomorphic inversion of Cactaceae, which in *O. quimilo* also includes the *trnV*^*UAC*^ gene. Regions I, IV, V, VI, and VII are colinear in both plastomes. Region II is colinear but is translocated in the *O. quimilo* plastome, while region III is inverted and translocated. Region V comprise the genes that are typically in the IR region but are translocated to the large single copy in *O. quimilo*. Genes highlighted in orange are those typically found in the SSC but transferred to the IR region in *O. quimilo*. Orange dashed line indicate the double inversion on the *ycf1-rpl32* genes, placing *ycf1* gene adjacent to *rpl32*. Black triangles represent duplicated genes present in purslane but absent in *O. quimilo*; LSC, large single-copy region; SSC, small single-copy region; IR, Inverted repeat.

The LSC of the *Opuntia quimilo* plastome appears to have experienced an expansion, with surprisingly 101 kb, while the SSC was shown to have exceptional reduction (4 kb). The LSC contains 24 tRNA genes and 67 CDS, and the SSC contains a unique tRNA gene (*trnL*^*UAG*^), and four CDS: *ccsA*, *ndhE*, *ndhD* and *psaC* (Figures 1, 2 and Tables 1, 2). A total of eight genes (*ndhB*, *rpl2*, *rpl23*, *rps7*, *rps19*, *trnI*^*CAU*^, *trnL^*CAA*^*, and *ycf2*) that are usually reported occurring in the IR regions of canonical angiosperm plastomes are apparently present as unique genes – not repeated – in the LSC region of the *O. quimilo* plastome (Figure 2, region V). On the contrary, seven genes (*ndhA*, *ndhF*, *ndhG*, *ndhH*, *ndhI*, *rpl32*, and *rps15*), usually from the SSC, are duplicated into the IR regions of the *O. quimilo* plastome (Figure 2, orange genes).

When compared to the canonical angiosperm chloroplast genome of *Portulaca oleracea*, two block translocations in the LSC are present in the *O. quimilo* plastome: the first (Figure 2, region II) is a simple colinear translocation of nine genes (Figure 2, region II); while the second one is a big block inversion and translocation comprising 50 genes within the *trnG*^*UCC*^-*psbE* region (Figure 2, region III). Inside that block (region III), the putative synapomorphic inversion of cacti encompassing the *trnM-rbcL* genes is confirmed for Cactaceae, but in the *O. quimilo* plastome this inversion also encompassed the *trnV*^*UAC*^ gene (Figure 2, green bars). Further gene order is mainly colinear (Figure 2, regions I, IV, V, VI, VII), except for the rearrangement comprising the SSC genes that were transferred to the IR regions, including a double inversion on the *ycf1-rpl32* region, placing *ycf1* gene adjacent to *rpl32* (Figure 2, orange genes).

### Reference-Guided Assemblies and Comparative Chloroplast Sequence Analyses

The reference-guided assembles of the remaining Opuntioideae and outgroup taxa to the *Opuntia quimilo* plastome (one inverted repeat stripped) mapped an average of 616,615 reads with a mean genome depth of 721x (Supplementary Table S2). The consensus sequence length varied between 126,925 bp [*Pereskiopsis diguetii* (F.A.C. Weber) Britton & Rose] to 129,181 bp [*Tacinga palmadora* (Britton & Rose) N.P. Taylor & Stuppy] and the GC content between 35.8% (*Pterocactus gonjianii* R. Kiesling) to 36.3% [*Austrocylindropuntia cylindrica* (Lam.) Backeb. and *Cylindropuntia bigelovii*] (Supplementary Table S2).

**FIGURE 3:**
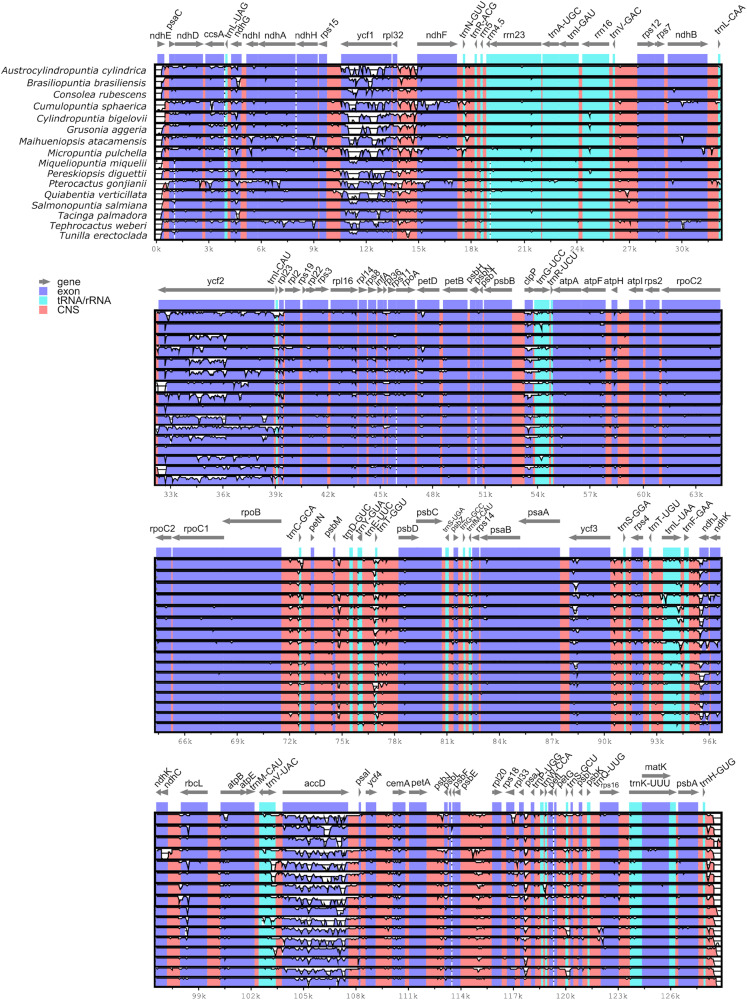
Visualized alignment of the Opuntioideae chloroplast genome sequences (one IR stripped) with annotations using mVISTA. Each horizontal lane shows the graph for the sequence pairwise identity with *Opuntia quimilo* as reference. The x-axis represents the base sequence of the alignment and the y-axis represents the pairwise percent identity within 50–100%. Gray arrows represent the genes and their orientations. Dark-blue boxes represent exon regions; light-blue boxes represent tRNA and rRNA regions; red boxes represent non-coding sequence (CNS) regions.

Pairwise comparison of divergent regions within the Opuntioideae chloroplast genome sequences using mVISTA with *O. quimilo* as a reference revealed both strikingly conserved and divergent regions across the chloroplast genome sequences (Figure 3). Overall, the alignment uncovered sequence divergence across assemblies, suggesting that chloroplast genome sequences are not conserved. Divergences were observed both in non-coding regions and coding regions. Among coding regions (CDS), non-conserved regions were frequent on genes of the *ndh* gene suite (i.e., *ndhA, ndhD, ndhE*, *ndhF*, *ndhG*, *ndhH, ndhI*, *ndhJ*) as well *clpP*, *ycf3* and particularly highlighted on *ycf1*, *ycf2*, and *accD* genes (Figure 3). Ten non-coding regions show substantial divergence, being all intergenic spacers: *ndhE-psaC, rpl32-ndhF, trnV^*GAC*^-rps12, psbB-clpP, rpoB-trnC^*GCA*^, psbM-trnD^*GUC*^, trnT^*GGU*^-psbD, psbE-rpl20, ndhC-rbcL* (Figure 3).

**FIGURE 4:**
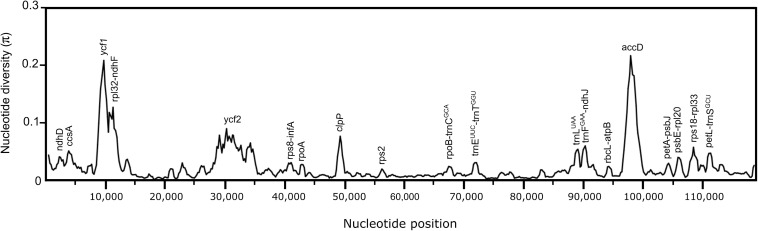
Nucleotide diversity graphs of the 17 Opuntioideae chloroplast genome sequences from the sliding windows analysis performed in DnaSP (windows length: 800 bp, step size: 200 bp). The x-axis represents the base sequence of the alignment, and the y-axis represents the nucleotide diversity (π value). Each variation hotspot for the chloroplast genome sequences of the Opuntioideae alignment is annotated on the graph.

The nucleotide diversity values (π) within the 17 Opuntioideae chloroplast genome sequences ranged from 0.00191 to 0.18551, with a mean value of 0.02201, indicating the sequences as highly variable. Three major regions were identified as hypervariable (π > 0.1), which comprises *ycf1* and *accD* genes and an intergenic spacer *rpl32-ndhF* (Figure 4); while six regions were observed as moderately-variable (π > 0.05), those being four genes: *ycf2*, *ccsA*, *clpP* and *trnL*^*UAA*^; and two intergenic spacers *rps18-rpl33* and *trnF^*GAA*^-ndhJ* (Figure 4).

**FIGURE 5:**
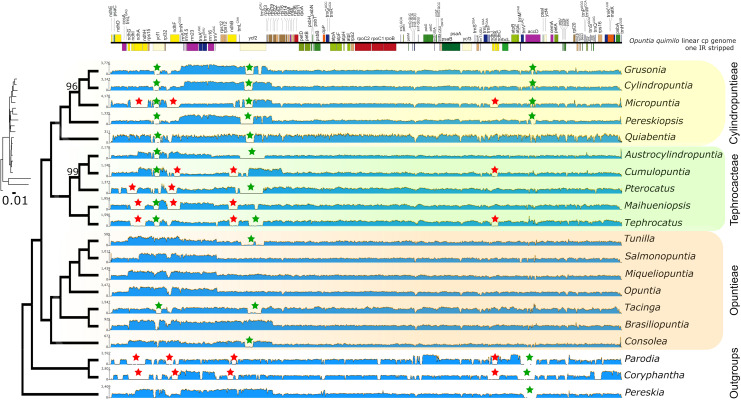
Maximum likelihood phylogenetic tree from RAxML analysis transformed in cladogram with the phylogram represented in small size with substitution rate scaled. All nodes have total bootstrap values (bs = 100) with exception for those that are shown above the branch. Each tip is represented with the assembly map of raw read coverages from Geneious mapper to the *Opuntia quimilo* chloroplast genome (one IR stripped, represented on the top with annotated genes). Red stars represent low coverage mapping and putative losses associated with the *ndh* gene suite; green stars represent partial low coverages associated with putative pseudogenization of *ycf1*, *ycf2*, and *accD* genes. Tribe Opuntieae is highlighted in orange, Tephrocacteae in green and Cylindropuntieae in yellow.

Reference-guided assembled maps of Opuntioideae and outgroups to the *Opuntia quimilo* chloroplast genome as a reference revealed regions with extremely low coverage or even gaps across different taxa (Figure 5). The regions highlighted with this feature are related with genes of the *ndh* suite, *ycf1*, *ycf2* and *accD*, suggesting gene loss, transfer to nuclear genomes and/or pseudogenization (Figure 5). Several members of Opuntioideae appear to have missing *ndh* genes in their chloroplast genome (*Micropuntia, Maihueniopsis, Pterocactus, Tephrocactus*), especially in the Tephrocacteae clade, but without a clear pattern across lineages.

### Phylogenetic Analyses and Informative Regions

**FIGURE 6:**
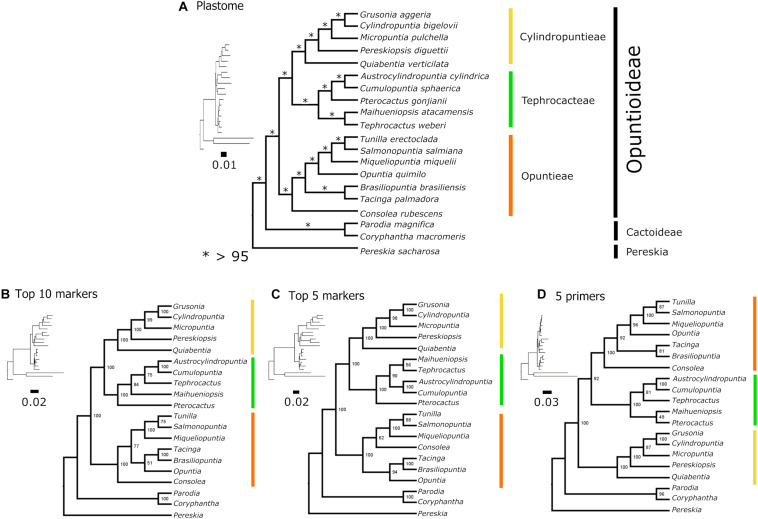
Topological comparisons of different datasets based on ML analyses. **(A)** Plastome dataset topology, **(B)** top 10 marker dataset topology, **(C)** top five marker dataset topology, and **(D)** five marker dataset topology for which primers were designed. The Cylindropuntieae sister to Tephrocacteae + Opuntieae topology was recovered only in the five-marker primer dataset **(D)**. Generic relationships are highly variable in Tephrocacteae among the datasets used. ^∗^bootstrap support.

The full chloroplast genome sequences resulted in an alignment of 118,930 bp with 86,484 identical sites (72.7%), a pairwise identity of 94.5% and 8,694 distinct alignment patterns. There were 8,922 parsimony informative sites (PIS) and 11,509 sites with gaps. Maximum Likelihood analyses resolved a well-supported Opuntioideae (bs = 100), with three major subclades (those currently circumscribed as tribes), Opuntieae, Cylindropuntieae and Tephrocacteae (Figure 6A). Opuntieae, consisting of the seven genera *Consolea*, *Brasiliopuntia*, *Tacinga*, *Opuntia, Miqueliopuntia*, *Salmonopuntia* and *Tunilla*, was resolved as sister to a Tephrocacteae (*Tephrocactus*, *Maihueniopsis*, *Pterocactus*, *Cumulopuntia*, and *Austrocylindropuntia*) + Cylindropuntieae (*Quiabentia*, *Pereskiopsis*, *Micropuntia*, *Grusonia*, and *Cylindropuntia*) clade. All nodes had full bootstrap support values (bs = 100), except at two nodes, which were still higher than 90% (Figure 6A).

The summary statistics for all markers (cpCDS, cpNCDS, cpIGS) are presented in Supplementary Table S3. A list of the top 10 markers ranked by phylogenetic information considering topological distance to the plastome tree, mean bootstrap support and number of parsimony informative sites is given in Table 3. All single marker phylogenies presented some disagreement to the plastome tree (RF tree distance ranging from 6 to 28), with bootstrap support ranging from 0 to 89 (mean = 37), and number of PIS from 0 to 619 (mean = 25), revealing many markers as not useful for phylogenetic inference (Supplementary Table S3). Phylogenetic trees of the top 10 individual markers are shown in Supplementary Figures S1, S2, and all trees are available as Supplementary Material. Primer pair sequences for PCR amplification are provided for the top five markers with suitable Sanger sequencing size (maximum ∼900 bp) in Table 4.

**TABLE 3:** Summary statistics for the top 10 markers.

	Bp	Aligned (bp)	Variable	PIS	Sites with gaps	Tree distance	Bootstrap (mean)
**(1) accD (cpCDS)**	1876 [1489–1927]	1953	966	586	616	10	88
**(2) ycf1 (cpCDS)**	1565 [1414–1615]	1650	958	429	456	8	76
**(3) ndhD (cpCDS)**	1421 [1410–1421]	1421	210	52	11	6	79
**(4) trnK^UUU^ (cpNCDS)**	2570 [2564–2572]	2573	173	45	15	8	82
**(5) psbE-rpl20 (cpIGS)**	1731 [1714–1736]	1739	242	68	83	8	77
**(6) petD (cpCDS)**	1265 [1257–1272]	1274	69	27	27	8	75
**(7) ccsA (cpCDS)**	1008 [1007–1011]	1011	110	49	4	8	73
**(8) clpP (cpCDS)**	359 [356–362]	362	112	64	6	8	70
**(9) rpoC2 (cpCDS)**	4101 [4101–4101]	4101	165	47	0	8	69
**(10) rpoC1 (cpCDS)**	2468 [2467–2469]	2469	86	35	4	8	69

*Markers are ranked by phylogenetic information based on a weighed mean of relative values of number of variable sites (weight = 1), mean bootstrap (weight = 2) and distance to the full plastid tree (weight = 3).*

**TABLE 4:** Primer pair sequences for the identified top 5 highly informative markers across the 17 chloroplast genome sequences of Opuntioideae.

Marker	Primer forward (5′–3′)	Primer reverse (5′–3′)	T_a_ (°C)	Expected product size (bp)
**psbB/clpP**	ACCAAGGCAAACCCATGGAA	TCCCCTTCTTACCAGCATCA	60	931
**ycf4-cemA**	GTCCTATTTCCTGCGTGTACCA	TGATAGAGAGATCCACCAGGGT	60	864
**rps2**	TTGAGATTCAGGAATAGTAACCGA	GTGTATCAATGGCCAATCCGC	57	885
**rbcL-atpB**	CAAAACAACAAGGTCTACTCGACA	GGAAACCCCAGGACCAGAAG	60	830
**petA**	ACGATTGATTGGACCATGCA	TCGGACAATTGAACCTTCTCGA	60	965

Phylogenetic inferences from the top 5 and top 10 markers concatenated yield similar topologies compared with the plastome tree, supporting three tribes (bs = 100) and Opuntieae as sister to (Tephrocacteae + Cylindropuntieae), although, there were minor incongruences within Tephrocacteae and Opuntieae (Figures 6B,C). Contrarily, phylogenetic inference from the five markers, which had primers designed (< ∼900 bp) revealed a conflicting topology, with Cylindropuntieae as sister to (Tephrocacteae + Opuntieae) with high support (bs = 92), and all three tribes with full support (bs = 100) (Figure 6D).

## Discussion

### Insights From Chloroplast Genome Assemblies in Opuntioideae and Cactaceae

The first chloroplast genome of a member of subfamily Opuntioideae and a species of *Opuntia* is here reported. Although the bulk of its gene content is not far from canonical angiosperm plastomes, it deviates in some cases from the typical chloroplast genome structure, showing: (i) an expansion of the LSC incorporating genes that are typically in the IRs; (ii) a reduction of the SSC translocating some common genes of the SSC into the IR region; and (iii) at least one massive translocation with an inversion of a block of genes in the LSC (Figure 2). Part of the content of the IRs in the *O. quimilo* plastome remained remarkably constant, including all four rRNA and five tRNA genes that are nearly universally reported in IRs of land plants (Mower and Vickrey, 2018). The GC content observed in the *O. quimilo* plastome is regular as expected based on other chloroplast genomes, being an AT rich organelle, with differences observed between coding/non-coding regions, where selection may be acting to preserve GC content for amino acid coding (Raubeson and Jansen, 2005; Downie and Jansen, 2015; Daniell et al., 2016).

Successive expansion–contraction events or even multiple contractions have been recurrently reported as one of the main ways of developing structural changes across angiosperm plastomes (Downie and Jansen, 2015; Daniell et al., 2016; Fonseca and Lohmann, 2017; Weng et al., 2017; Mower and Vickrey, 2018) and may also be one way in which genes are translocated to different regions of the genome, as suggested in adzuki bean (Perry and Wolfe, 2002). The atypical reduction of the SSC (∼4 kb), reported here for the *O. quimilo* plastome, has also been noticed in *Viviana marifolia* (Francoaceae, Geraniales), and a slightly similar reduced size for the SSC (∼6 kb) have been inferred for the ancestral chloroplast genome of Geraniaceae (Weng et al., 2014). A partial deletion of the SSC region has also been reported in two hemiparasitic *Taxillus* (Loranthaceae) species resulting in a ∼6 kb region with only two genes (Li et al., 2017), and the smallest SSC hitherto reported is for the hemiparasitic *Pedicularis ishidoyana* (Orobanchaceae), with only 27 bp (Cho et al., 2018). A model to explain the major rearrangements observed in the *Lamprocapnos spectabilis* (Papaveraceae) plastome, involving at least six IR boundary shifts and five inversions resulting in a SSC of just 1,645 bp with a partial *ndhF* gene, was recently provided by Park et al. (2018). The SSC contains most *ndh* genes, and previous studies have shown that boundary shifts of the IR and SSC regions are correlated with transformations of *ndhF* and *ycf1* genes (Logacheva et al., 2014; Kim et al., 2015; Li et al., 2017).

The *Opuntia quimilo* plastome reinforces some different putative structural synapomorphies that have been reported in Caryophyllales. The loss of the *rpl2* intron, previously suggested to be absent throughout the Centrospermae (Palmer et al., 1988), is supported in our study and other newly assembled plastomes in Caryophyllales (Yao et al., 2019). The *trnM-rbcL* inversion is again recovered in the *O. quimilo* plastome, although also involving the *trnV*^*UAC*^ gene, as in *Cylindropuntia bigelovii* (Majure et al., 2019), providing further support for this inversion as a synapomorphy in the family. Additionally, Sanderson et al. (2015) and Solórzano et al. (2019), inspecting plastomes of Cactoideae, reported a gene orientation of *ycf2-trnL*^*CAA*^-*ycf1* in the SSC as a synapomorphy of Cactoideae. Our results corroborate this observation, since this feature is not present in the *O. quimilo* plastome, strengthening this gene order as a putative synapomorphy for Cactoideae. On the other hand, the *ycf1-rpl32-ndhF* orientation, reported in the *Cylindropuntia bigelovii* chloroplast sequence (Majure et al., 2019), is recovered in the *O. quimilo* plastome and is here suggested as a putative synapomorphy for Opuntioideae.

Reference-guided assemblies and comparative analyses revealed insights for plastome rearrangements across disparate Opuntioideae. The differences of depth and coverage among specific chloroplast genes suggest that gene presence or structure may vary over species in Opuntioideae, as have been observed in other Cactaceae, specifically Cactoideae (Sanderson et al., 2015; Solórzano et al., 2019). The putative independent losses of several *ndh* genes in all Cactoideae plastomes assembled hitherto, such as the saguaro cactus and several *Mammillaria* species, can be also inferred for our Cactoideae outgroups sampled (*Parodia magnifica* and *Coryphantha macromeris;* Figure 5, red stars). Likewise, some members of Cylindropuntieae and Tephrocacteae (*Micropuntia*, *Cumulopuntia*, *Pterocactus*, *Maihueniopsis*, and *Tephrocactus*) also likely experienced independent losses of several genes of the *ndh* suite in their chloroplast genomes, although this was not so for tribe Opuntieae, where those genes were found to be intact (Figure 5, red stars), indicating putative homoplasious events. The putative loss of one of the inverted repeat regions in *Quiabentia* must be further investigated through rigorous *de novo* assemblies (Figure 5).

Loss of *ndh* genes or the *ndh* gene suite has been reported in both gymnosperms (Wakasugi et al., 1994; McCoy et al., 2008; Wu et al., 2009) and angiosperms, as well as some other photosynthetic organisms. The loss of such genes is well-known and is often associated with hemi- or holoparasitism where genes necessary for photosynthesis are often unessential (e.g., *Epifagus*, Orobanchaceae, De Pamphilis and Palmer, 1990; Santalales, Shin and Lee, 2018). However, a number of autotrophic plants have also shown a similar trend with losses or pseudogenization of *ndh* genes. For example, Lin et al. (2017) showed the repeated loss of *ndh* genes across several different autotrophic orchid species and suggested that those losses could have been a step toward heterotrophy. Ruhlman et al. (2015) suggested that the evolution and retention of the NDH (NADH dehydrogenase-like) complex was associated with the transition of plants to environmentally stressful environments, and that *ndh* gene loss may be associated with a relaxed reliance on the complex based on decreased environmental stressors (e.g., through reliance on host species for resources in parasites).

Contrastingly, there are numerous reports of *ndh* loss or pseudogenization in angiosperms associated with the presence of CAM photosynthesis, which has evolved as a response to water limited habitats (i.e., water stress), such as in desert or other edaphically arid areas where cacti occur or also associated with an epiphytic habit, for instance in orchids (Luo et al., 2014; Givnish et al., 2015; Sanderson et al., 2015). Whether or not the absence of those *ndh* genes in the chloroplast corresponds to their integration into the nuclear genome often remains to be determined, but there are some studies showing that those genes likewise, have not been incorporated into the nucleus (Lin et al., 2017) and thus are totally lost. Certain species of Opuntioideae have been shown to be facultatively CAM species (Winter et al., 2011), whereas other species appear to be obligate CAM. Perhaps the putative loss or pseudogenization of *ndh* genes across members of Opuntioideae coincides with the transition to more water limited habitats and thus a stronger association with obligate CAM photosynthesis. Although assumed that most derived cacti (Cactoideae, Opuntioideae) are obligate CAM, there are actually very little data to show photosynthetic pathways across Cactaceae, and the retention of large leaves in Opuntioideae bring into question this assumption (Majure et al., 2019). Likewise, our knowledge of CAM photosynthesis is in a state of flux, and it is clear that there are taxa that do not clearly fit into basic photosynthetic pathways as traditionally defined (Edwards, 2019). The putative connection with *ndh* gene loss and CAM photosynthesis needs to be rigorously tested.

The major plastid regions marked by pseudogenization in the *Opuntia quimilo* plastome (*ycf1*, *ycf2*, and *accD*) are visually highlighted as non-conserved regions in reference-guided maps (Figure 5, green stars), as in the mVista alignment across Opuntioideae (Figure 3). These regions are also emphasized as with hyper or moderate variability regarding nucleotide diversity values (Figure 4). All genes here reported as pseudogenes in the *O. quimilo* plastome (*accD*, *ycf1*, and *ycf2*) have also been reported as pseudogenes in the *Mammillaria* plastomes (Solórzano et al., 2019), while the *accD* was described as a pseudogene in *Carnegiea gigantea* (Sanderson et al., 2015). Pseudogenization of these genes has been repeatedly reported across different angiosperm lineages, such as Malpighiales, Campanulales, Ericales, Poales, Solanales, Geraniales, Santalales, and Myrtales (Haberle et al., 2008; Fajardo et al., 2013; Harris et al., 2013; Weng et al., 2014; Li et al., 2017; Machado et al., 2017; Bedoya et al., 2019; Cui et al., 2019). Even though these genes have been identified with essential functions beyond photosynthesis and retained in the plastome of most embryophytes (Drescher et al., 2000; Kuroda and Maliga, 2003; Kode et al., 2005; Kikuchi et al., 2013; Parker et al., 2014; Dong et al., 2015), there are several other plants where these genes are missing from the chloroplast genome (Magee et al., 2010; Kim et al., 2014; Liu et al., 2016; Graham et al., 2017). The pseudogenization or loss of the *accD*, *rpl22* and several genes of the *ndh suite* from the plastids has been reported to be a consequence of them being transferred to the nuclear genome (Jansen et al., 2011; Jansen and Ruhlman, 2012; Sanderson et al., 2015; Liu et al., 2016; Cauz-Santos et al., 2017). Plastid gene transfer to the nucleus remains to be examined in *O. quimilo* and related Opuntioideae.

Several regions highlighted as hyper or moderately variable regarding the nucleotide diversity values across Opuntioideae chloroplast sequences (i.e., *accD*, *ycf1*, *clpP, petD, rpl32*, and *ccsA*) have been reported to be putatively under positive selection in some lineages, such as Brassicaceae, Bignoniaceae, Rutaceae, Orchidaceae, Geraniaceae, and Poaceae (Carbonell-Caballero et al., 2015; Hu et al., 2015; Weng et al., 2016; Park et al., 2017; Dong et al., 2018; Piot et al., 2018; Ruhlman and Jansen, 2018; Thode and Lohmann, 2019). Positive selection may come into play in response to environmental changes (Piot et al., 2018). For example, the *accD* gene, which encodes the β-carboxyl transferase subunit of acetyl-CoA carboxylase, is an essential and required component for plant leaf development (Kode et al., 2005), and it is suggested to have played a pivotal role in the adaptive evolution of orchids (Dong et al., 2018). The signatures of positive selection in the *accD* gene observed in Brassicaceae and Campanulaceae have also indicated that this gene may have been repeatedly involved in the adaption to specific ecological niches during the radiation of eudicotyledonous plants (Rousseau-Gueutin et al., 2013; Hu et al., 2015). Considering the harsh environment that cacti occupy, their fitness already expressed in its peculiar morphology and physiology, further studies should be carried out to investigate the putative relation of chloroplast rearrangement – such as pseudogenization, loss of genes, translocation and inversion – with ecological features.

### Phylogenetic Relationship of Opuntioideae Tribes

The plastome phylogeny of Opuntioideae strongly resolves three major and well-supported clades, the tribes Opuntieae (O), Tephrocacteae (T) and Cylindropuntieae (C) (Figure 6A). Three previously described tribes (Austrocylindropuntieae, Pterocacteae, and Pereskiopsideae) (Doweld, 1999; Wallace and Dickie, 2002), mainly representing lineages of a single genus, are nested within these more broadly circumscribed tribes, and thus have no real practical taxonomic use (Hunt, 2011).

In our phylogenomic analyses, Opuntieae was sister to a Tephrocacteae/Cylindropuntieae clade, as in Majure et al. (2019), who also used plastome data but with a reduced taxon sampling in Opuntieae and Tephrocacteae. This same topology [O + (T + C)] was further uncovered with high support using our top 10 and 5 phylogenetic informative markers concatenated (Figures 6B,C). On the other hand, Walker et al. (2018) and Wang et al. (2019), using transcriptome data, revealed Cylindropuntieae as sister to an Opuntieae/Tephrocacteae clade [C + (O + T)] yet with very limited taxon sampling. Likewise, this alternate topology [C + (O + T)] was recovered in our study when exploring our five markers concatenated, which have primer-pairs designed (Figure 6D), i.e., those best ranked markers with suitable size for PCR amplification (< ∼900 bp) (see further discussion in the next section). Hernández-Hernández et al. (2011, 2014), as well as Bárcenas et al. (2011), although recovering the same three tribes sampling few genera, did not resolve the relationships among them, while Nyffeler (2002) did not have sufficient taxon sampling to infer relationships within Opuntioideae. Thus, this recalcitrant relationship between the three tribes must be further investigated using more genealogies, such as nuclear, plastome and mitochondrial datasets.

Griffith and Porter (2009) previously tackled relationships within Opuntioideae using DNA sequence data with a comprehensive sampling, yet based only on nrITS and *trnL-trnF* data. Our results partially recovered their topology, with a “flat-stemmed” and a “terete-stemmed” clade, moderately equivalent to our Opuntieae and Cylindropuntieae tribes, respectively. However, many members of Tephrocacteae recovered here were nested within their “terete-stemmed” clade, such as *Austrocylindropuntia, Cumulopuntia* and *Tephrocactus.* Likewise, the Griffith and Porter (2009) topology revealed a grade of two clades (*Pterocactus* and *Maihueniopsis*), which were sister to the rest of Opuntioideae but that was not recovered in our study. However, as our study is still based on one sample per genus, future studies including a wider sampling should be carried out across the subfamily to further test the relationships here recovered and are currently underway (Majure et al., in preparation).

Tribe Opuntieae is the most diverse and widespread clade among Opuntioideae, consisting of seven accepted genera and around 230 species (Majure and Puente, 2014; Guerrero et al., 2019). *Consolea* Lem., an endemic tree-like cactus of the Caribbean Islands and neighboring areas (Majure et al., submitted), is sister to the rest of Opuntieae, which consists of two subclades: (i) one comprising *Brasiliopuntia* (K. Schum.) A. Berger + *Tacinga* Britton & Rose; and the other comprising (ii) *Opuntia* (L.) Mill. + [*Miqueliopuntia* Friè ex F. Ritter + (*Salmonopuntia* P.V. Heath + *Tunilla* D.R. Hunt & Iliff.)]. Previous analyses did not resolve this position for *Consolea*, mostly based on the lack of data/data type (Griffith and Porter, 2009) and/or outgroup taxon sampling (Majure et al., 2012; Majure and Puente, 2014). Likewise, the sister relationship of *Opuntia* with the (*Salmonopuntia* + *Tunilla*) + *Miqueliopuntia* clade had not been recovered in previous analyses (Majure et al., 2012).

Based on our plastome analysis, Cylindropuntieae and Tephrocacteae are sister tribes, comprised of five and six genera, respectively (Figure 6A). Cylindropuntieae are primarily represented by genera that occur in the western North American desert regions [*Cylindropuntia* (Engelm.) F.M. Knuth, *Grusonia* F. Rchb. & K. Schum. and *Micropuntia* Daston], which formed a well-supported subclade, but they also contain two genera that are found in tropical dry forest of Mexico/Northern Central America (*Pereskiopsis* Britton & Rose) and Tropical Dry Forest and Chaco of South America (*Quiabentia* Britton & Rose). Tribe Pereskiopsideae (Doweld, 1999), previous described to only accommodate the leafy *Pereskiopsis*, is nested within Cylindropuntieae and is redundant, and thus unnecessary. Deeper relationships within Cylindropuntieae were recently untangled using a phylogenomic approach and dense sampling, revealing biogeographic patterns as well as characters evolution (Majure et al., 2019). Our plastome phylogeny here revealed an identical phylogenetic pattern among genera (Figure 6A) of Majure et al. (2019), and equivalent to Bárcenas (2016).

Tephrocacteae is a South American clade adapted to diverse climatic conditions over a wide area of the southern Andes and adjacent lowlands (Ritz et al., 2012; Guerrero et al., 2019; Las Peñas et al., 2019). The tribe includes morphologically diverse species from geophytes and cushion-plants to dwarf shrubs, shrubs or small trees (Anderson, 2001); and probably geophytes and cushion-forming species evolved several times from shrubby-like precursors (Ritz et al., 2012). Tribes Austrocylindropuntieae and Pterocacteae (Wallace and Dickie, 2002) were described to circumscribe *Austrocylindropuntia* + *Cumulopuntia* and *Pterocactus*, respectively, and both are nested within the Tephrocacteae as amplified by Hunt (2011). So, as shown here, their use is mostly redundant. Although our plastome data recovered *Maihueniopsis* and T*ephrocactus* as sister to *Pterocactus* + (*Austrocylindropuntia* + *Cumulopuntia*), the phylogenetic topology among genera of the tribe are highly variable when using different datasets (Figures 6A–D). It is probable that increased taxon sampling may ameliorate this topological variability, as we still lack whole plastome data for the monospecific genus *Punotia*. Greater taxon and data sampling will be necessary to fully test these relationships.

### Phylogenetically Informative Regions

Our plastome survey for phylogenetically informative markers revealed a list of potentially highly informative plastid markers for Sanger-based phylogenetic studies in Opuntioideae (Supplementary Table S3). The top 10 markers in our cpCDS dataset are: *accD, ycf1*, *ndhD, petD, ccsA, clpP, rpoC1, rpoC2*, including just one intron (the *trnK* intron comprising the *matK* gene – *trnK/matK*) and one intergenic spacer (*psbE-rpl20*) (Table 3). However, two of the better ranked markers (*accD* and *ycf1*) are putative pseudogenes and must be treated apart from traditional protein coding genes. The impact and utility of pseudogenes as markers for phylogenetic inferences must be further investigated (see below).

From the top 10 markers ranked in our list, just one (*trnK/matK*) has been used in more than one phylogenetic study in cacti (Nyffeler, 2002; Edwards et al., 2005; Korotkova et al., 2010; Arakaki et al., 2011; Bárcenas et al., 2011; Demaio et al., 2011; Hernández-Hernández et al., 2011, 2014; Ritz et al., 2012; Bárcenas, 2016; Vargas-Luna et al., 2018); while Majure et al. (2012) and Franck et al. (2013) used partial sequences of the *ycf1* gene. The other top 10 markers have been previously used under phylogenomic approaches in cacti (Arakaki et al., 2011; Majure et al., 2019).

Although the majority of the top 10 markers here reported have not been used in phylogenetic studies of cacti, the relationship of several other groups has been inferred with some of these markers. For example, the *accD* gene, combined with other plastid regions including *rpoC1*, was employed for phylogenetic inference of *Crocus* (Iridaceae), *Coptis* (Ranunculaceae) and Orchidaceae genera (Petersen et al., 2008; Guo et al., 2012; He et al., 2014). However, *accD* intergenic spacers, such as *rbcL-accD* and *accD-psaI*, have been much more widely used across disparate groups (Barfuss et al., 2005; Miikeda et al., 2006; Reginato et al., 2010; Sun et al., 2012; Michelangeli et al., 2013). The *ycf1* gene appears to be moderately used (Gernandt et al., 2009; Guo et al., 2012; Majure et al., 2012; Shi et al., 2013; Whitten et al., 2013; Dastpak et al., 2018), and increasingly reported to be a useful marker in phylogenetic inferences (Neubig et al., 2009; Neubig and Abbott, 2010; Dong et al., 2012; Thomson et al., 2018), and the most promising plastid DNA barcode of land plants (Dong et al., 2015). The *petD* intron has been used (Löhne et al., 2007; Worberg et al., 2007; Borsch et al., 2009; Scataglini et al., 2014), but in our analysis the entire gene was used (exon + intron) showing phylogenetic utility. The *ccsA* gene seems to be underexplored as a phylogenetic marker (Marx et al., 2010; Peterson et al., 2012) but was already suggested as convenient for phylogenetic inferences (Logacheva et al., 2007). The *rpoC1* and *rpoC2* genes have been occasionally used together as markers (Liston and Wheeler, 1994; Kulshreshtha et al., 2004) or combined with other markers (Downie et al., 2000; GPWS, 2001; Zhang et al., 2011; Guo et al., 2012) yielding satisfactory results. The *rpoC2* gene was recently found as the best performing marker to recover, with high levels of concordance, the “accepted tree” of the angiosperm phylogeny (Walker et al., 2019). The *ndhD* gene seems to be scarcely used for phylogenetic inference (Panero and Funk, 2002), while the intergenic spacer of *psbE-rpl20* genes has never been used individually to our knowledge.

Eight of the top 10 markers are more than 900 bp, indicating that longer genes are superior for phylogenetic reconstruction, as previous suggested by Walker et al. (2019), although they may require internal primer designing for complete Sanger sequencing. A list of the top 10 markers with less than 900 bp is reported (Supplementary Table S4), and primer pair design for the top five is provided in Table 4. Our phylogenetic inference from the top five markers concatenated, which had primers designed (Figure 6D) recovered a conflicting topology compared with the plastome tree (Figure 6A). The topology with Cylindropuntieae as sister to Tephrocacteae + Opuntieae has also been recovered based on transcriptome data (Walker et al., 2018; Wang et al., 2019). Curiously, we obtained this same topology, although not strongly supported, using the top 10 marker dataset concatenated, when stripping the two pseudogenes *accD* and *ycf1* (Supplementary Figure S3), suggesting that functional constraints of these pseudogenes may influence the underlying topology.

Our top five markers contained intergenic spacers, which influence our alignment, wherein the incorporation of gaps is necessary. Duvall et al. (2020) found that as gaps increased in their alignment of plastomes across Poaceae, differing topologies were increasingly supported. This may also play a role in the incongruent topologies recovered in our analyses. Perhaps a higher level of homoplasy across datasets including gaps may reduce their suitability for resolving deep phylogenetic relationships, however, those same regions (i.e., intergenic spacers) are likely more appropriate for resolving species relationships among closely-related species (Shaw et al., 2005). Likewise, selective pressures on the genes in both our reduced 10 marker datasets, as well as in previously published transcriptome data (Walker et al., 2018), may likewise influence topology. Homoplasy in these reduced datasets may also be a factor leading to conflicting topologies. More research should be focused on the level of utility of specific gene regions (e.g., coding genes, intergenic spacers) across clades.

Chloroplast markers have been used for testing evolutionary relationships among plants for the past 30 years (Gitzendanner et al., 2018). While the assumption that these markers are evolving as a single unit without recombination, routine analyses have used concatenated data producing highly supported phylogenies that have been underlying the current classification of angiosperms (APG, 2016). However, as here reported, no marker as a single unit (gene tree) recovered the same topology of the plastome inference (concatenated tree), and even within the top 10 markers listed, some showed high values of discordance (Table 3, Supplementary Table S4, and Supplementary Figures S1, S2). Such results discourage and call attention to phylogenetic approaches based on one or few markers. While the full chloroplast sequences showed to be the most robust dataset to resolve relationships within Opuntioideae, phylogenies from the top 10 and 5 markers concatenated resolved many relationships with high bootstrap values and few nodes with low support (Figures 6B,C). Although we cannot test how effective these datasets would work in determining closely related species relationships, based on our limited taxon sampling here, it is significant that these smaller datasets resolve relationships among these clades and genera that have not been resolved previously using a similar number of loci (e.g., Hernández-Hernández et al., 2011). Thus, we would expect that using these more highly variable loci, although few, should greatly increase resolution across many subclades in Cactaceae. We also encourage their use across subclades within Cactoideae to test their broader utility.

Recent studies have explored gene tree conflict in plastome-inferred phylogenies and incongruence between gene trees and species trees in plastid genes (Gonçalves et al., 2019; Walker et al., 2019). Gonçalves et al. (2019) emphasized the importance of considering variation in phylogenetic signal across plastid genes and the exploration of plastome data to increase accuracy of estimating relationships; they also revealed that phylogenies inferred with multispecies coalescent (MSC) methods are accurate with plastome matrices and should be considered in future phylogenomic investigations. Walker et al. (2019) highlighted that most plastid genes are largely uninformative and are unlikely to misguide plant systematics. However, the concatenating of plastid genes without some level of scrutiny can mislead branch length estimation (Walker et al., 2019). The causes of discordant topologies across gene trees from chloroplast genome still needs to be better investigated. The main explanations include systematic errors (e.g., poor modeling, stochastic events) or more biologically meaningful processes, such as heteroplasmic recombination that have been invoked to explain discordance in disparate plant clades (Huang et al., 2001; Marshall et al., 2001; Bouillé et al., 2011; Walker et al., 2019).

## Conclusion

Chloroplast genomes have long been considered conserved among land plants, but recent generation of 1000s of plastomes through NGS has illuminated that this is not always the case. Cactaceae are no exception to variation that has been observed in other clades. Previous plastomes of cacti have shown to have lost one copy of the inverted repeat regions and several genes of the *ndh* gene suite, as well as to possess divergent inverted repeat regions and the smallest chloroplast genome known for an obligately photosynthetic angiosperm. We showed that the *Opuntia quimilo* plastome also presents deviations of canonical angiosperm plastomes with an expansion of the LSC incorporating genes that are typically in the IRs, a reduction of the SSC translocating some common genes of the SSC into the IR region, and one massive translocation with an inversion of a block of genes in the LSC. Strikingly different from other cacti, two copies of the inverted repeat region were recovered in the *Opuntia quimilo* plastome. Our reference-guided assemblies across Opuntioideae allowed us to infer putative independent losses of some *ndh* genes across disparate taxa of the subfamily. We did not find synapomorphic plastome features within Opuntioideae clades, thus, we hypothesize that putative rearrangements across the subfamily are from homoplasious events. Further analyses should be carried out to address how ecological drivers and morphological traits of cacti may be related with positive selection of genes and the common rearrangements in chloroplast genomes that have been reported in the family. Phylogenetic analyses of chloroplast genome sequences strongly support Opuntioideae and its three tribes: Opuntieae, Cylindropuntieae, and Tephrocacteae. As computational and budget limitations are still a bottleneck to deal with high throughput data, especially in developing countries, a list of highly informative plastid markers is presented for future use, and several top ranked markers have not been used in phylogenetic studies of cacti. However, conflicting topologies were recovered among major clades when exploring different assemblies of markers, revealing that gene tree discordance among markers must be carefully considered while inferring phylogenies in this remarkable group of plants, especially considering the occurrence of putative pseudogenes. Even so, topological incongruences may actually signal deeper phylogenetic patterns underlying biologically relevant evolutionary history and should be further explored using both nuclear and plastome datasets.

## Data Availability Statement

The datasets generated for this study can be found in the GenBank: MN114084 and MT369928–MT369946.

## Author Contributions

MK: conceptualization, methodology, validation, formal analysis, investigation, data curation, writing – original draft, writing – review and editing, visualization, project administration, and funding acquisition. MR: methodology, software, validation, formal analysis, and writing – review and editing. TS-C: writing – review and editing. LM: methodology, validation, investigation, resources, data curation, and writing – review and editing, visualization, supervision, project administration, and funding acquisition.

## Conflict of Interest

The authors declare that the research was conducted in the absence of any commercial or financial relationships that could be construed as a potential conflict of interest.

## Footnotes

1 http://rapid-genomics.com/home/
