# Supplementary material for: Insights Into Chloroplast Genome Evolution Across Opuntioideae (Cactaceae) Reveals Robust Yet Sometimes Conflicting Phylogenetic Topologies
Source: Front Plant Sci. 2020 Jun 19;11:729. doi: 10.3389/fpls.2020.00729 (PMC7317007; doi:10.3389/fpls.2020.00729)
Supplement: Supplementary file 1 [file Data_Sheet_1.docx]

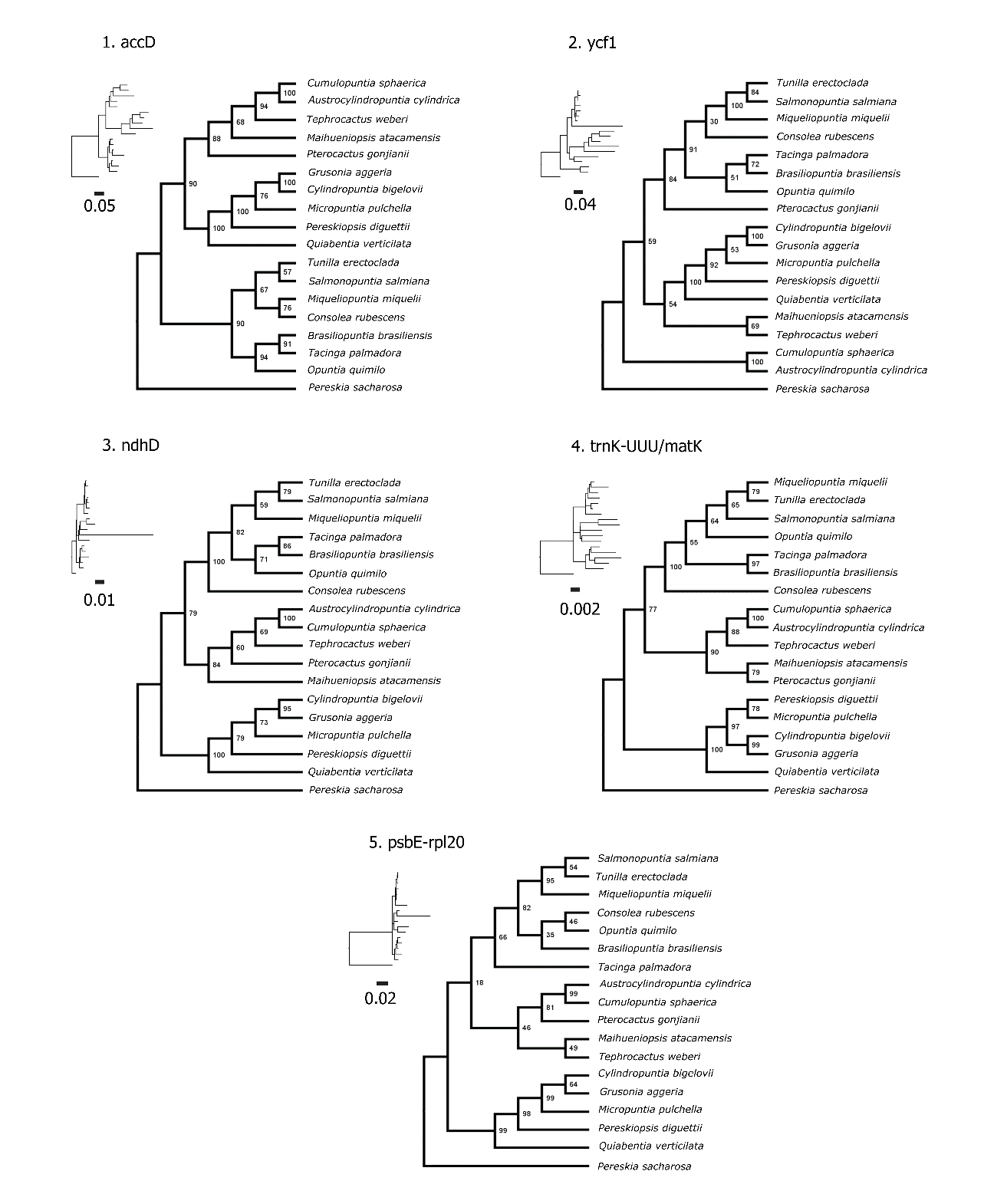
Supplementary Material

Supplementary Figure 1. Maximum likelihood phylogenetic tree from RAxML analyses based on individual top 10 markers, transformed in cladogram with the phylogram represented in small size with substitution rate scaled.


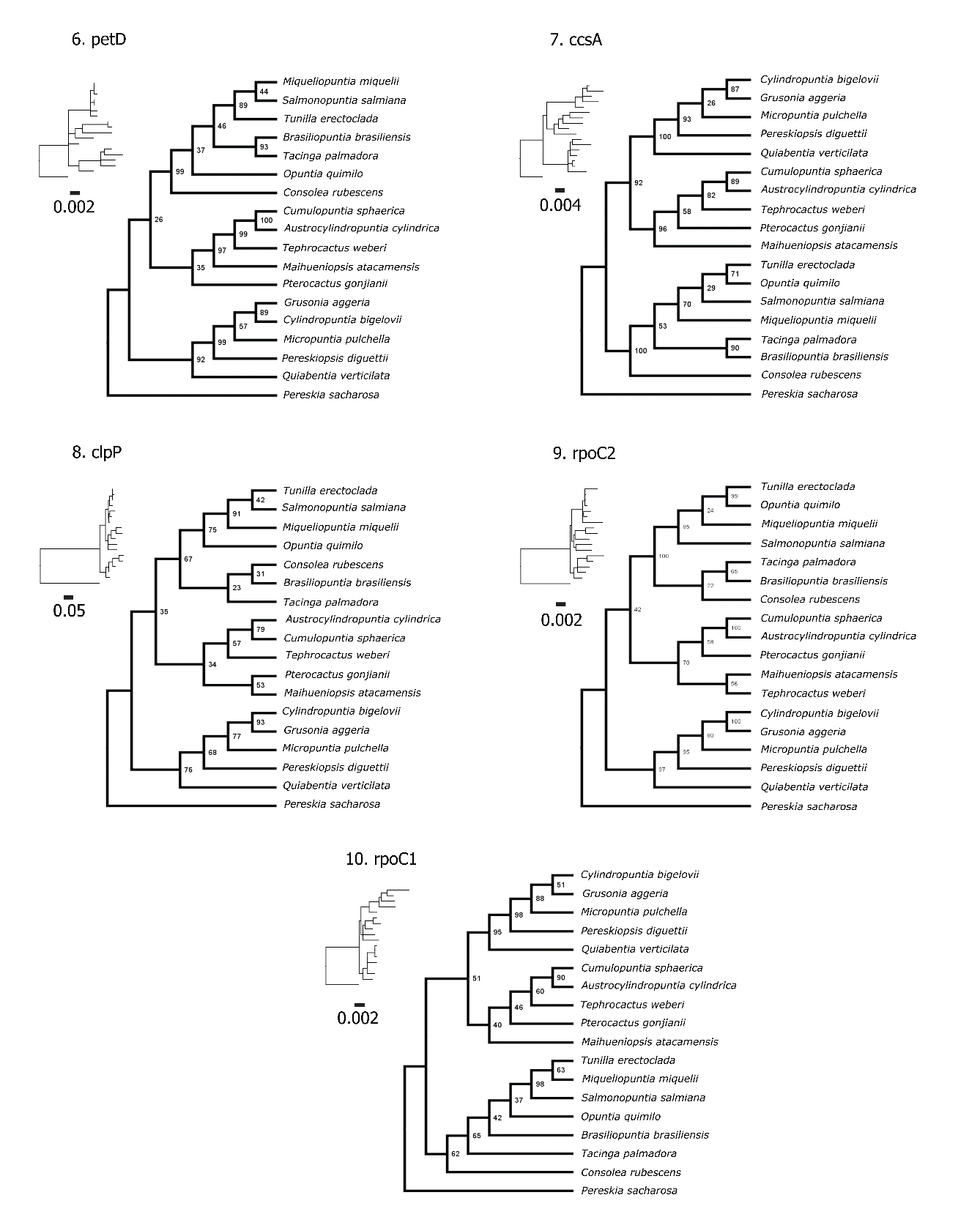


Supplementary Figure 2. Maximum likelihood phylogenetic tree from RAxML analyses based on individual top 10 markers, transformed in cladogram with the phylogram represented in small size with substitution rate scaled.


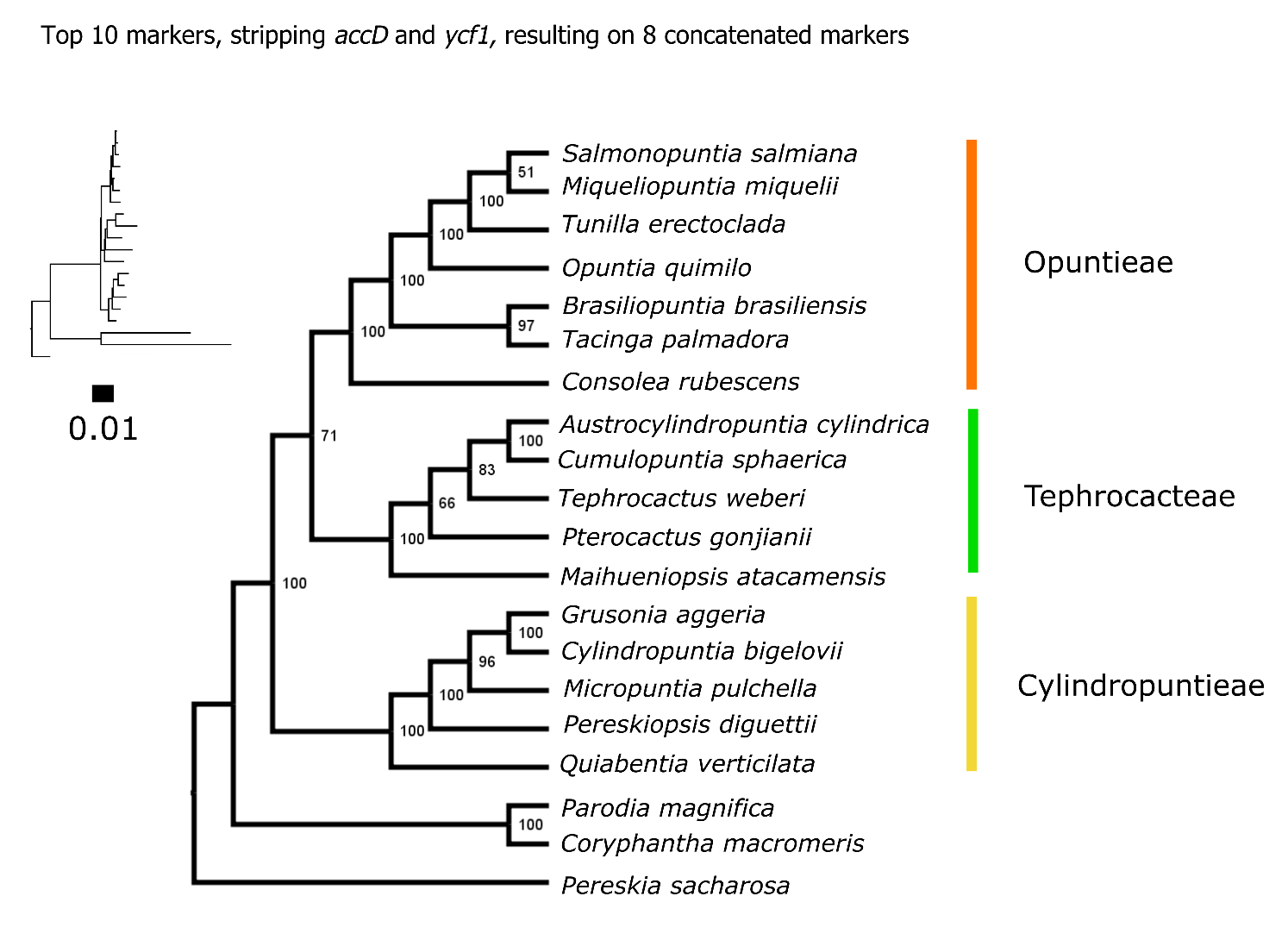


Supplementary Figure 3. Maximum likelihood phylogenetic tree from RAxML analysis based on top 10 markers concatenated, stripping pseudogenes *accD* and *ycf1.*

**Table S1.** Taxa sampled in this study, raw and post–quality control read numbers, GC content and voucher information. Vouchers contain collector name, number and herbarium repository in parentheses or the accession number from the Desert Botanical Garden Living collection (https://dbg.org/research-conservation/living-collections/).

| Taxon | Raw Read Nº | Read Nº Post-QC | %GC | Voucher |
| --- | --- | --- | --- | --- |
| *Austrocylindropuntia cylindrica* | 7724354 | 7362548 | 37.4 | *Baker19306* (ASU) |
| *Brasiliopuntia brasiliensis* | 5624110 | 5360990 | 39.6 | *Köhler 412* (ICN) |
| *Consolea rubescens* | 6227612 | 6038548 | 37.4 | *Majure 3320* (FLAS) |
| *Coryphantha macromeris* | 8294236 | 7997166 | 40.6 | *Majure 7128* (FLAS) |
| *Cumulopuntia sphaerica* | 8245847 | 8106104 | 37.6 | DBG1993-0190 |
| *Cylindropuntia bigelovii* | 16876574 | 16431880 | 39.2 | *Baker 18286* (ASU) |
| *Grusonia aggeria* | 16876574 | 16084111 | 39.5 | *Majure 5651* (DES) |
| *Maihueniopsis atacamensis* | 17489908 | 17322547 | 37.4 | DBG1993-0194 |
| *Micropuntia pulchella* | 20219350 | 19863298 | 39 | *Majure 6095* (DES) |
| *Miqueliopuntia miquelii* | 15182404 | 15065674 | 37.6 | DBG1993-0205 |
| *Opuntia quimilo* | 16137088 | 15814820 | 38.5 | DBG2011-0114 |
| *Parodia magnifica* | 5792409 | 5786100 | 38.3 | DBG1993-0275 |
| *Pereskia sacharosa* | 8781612 | 8526372 | 38.4 | DBG2014-2153 |
| *Pereskiopsis diguettii* | 8789752 | 8650470 | 38 | DBG2018-0128 |
| *Pterocactus gonjianii* | 9941712 | 9809046 | 38.9 | DBG1997-0260 |
| *Quiabentia verticillata* | 9223832 | 8893090 | 38.1 | *Kimnach 2803* (HNT) |
| *Salmonopuntia salmiana* | 6667776 | 6425092 | 38.8 | *Köhler 515* (ICN) |
| *Tacinga palmadora* | 18511428 | 18182592 | 39.2 | DBG1997-0392 |
| *Tephrocactus weberi* | 13461630 | 13300339 | 37.8 | DBG2012-0428 |
| *Tunilla erectoclada* | 6935606 | 6675910 | 37.8 | DBG2018-0144 |

**Table S2.** Reference-guide assemblies of Opuntioideae taxa to the *Opuntia quimilo* plastome, with the number of reads mapped to the *Opuntia quimilo* plastome, genome coverage, consensus sequence length (bp), GC content (%) and GenBank accession.

| Taxon | Chloroplast | | | | |
| --- | --- | --- | --- | --- | --- |
|  | Reads Mapped | Genome Coverage | Consensus Sequence Length | %GC | GenBank Submission |
| *Austrocylindropuntia cylindrica* | 551,356 | 642 | 127,734 | 36.3 | MT369946 |
| *Brasiliopuntia brasiliensis* | 386,272 | 449 | 128,560 | 36.1 | MT369945 |
| *Consolea rubescens* | 191,291 | 221 | 128,464 | 36.2 | MT369944 |
| *Coryphantha macromeris* | 817,912 | 982 | 128,341 | 36.2 | MT369943 |
| *Cumulopuntia sphaerica* | 333,795 | 393 | 127,844 | 36 | MT369942 |
| *Cylindropuntia bigelovii* | 1,159,529 | 1355 | 128,307 | 36.3 | MT369941 |
| *Grusonia aggeria* | 1,307,477 | 1534 | 127,410 | 36.2 | MT369940 |
| *Maihueniopsis atacamensis* | 661,251 | 775 | 128,476 | 36.2 | MT369939 |
| *Micropuntia pulchella* | 1,189,650 | 1404 | 127,876 | 36.1 | MT369938 |
| *Miqueliopuntia miquelii* | 544,818 | 638 | 128,543 | 36.1 | MT369937 |
| *Parodia magnifica* | 810,368 | 954 | 127,406 | 36.2 | MT369936 |
| *Pereskia sacharosa* | 919,347 | 1075 | 128,319 | 35.9 | MT369935 |
| *Pereskiopsis diguettii* | 449,688 | 523 | 126,925 | 36 | MT369934 |
| *Pterocactus gonjianii* | 481,070 | 552 | 127,885 | 35.8 | MT369933 |
| *Quiabentia verticillata* | 170,524 | 197 | 128,319 | 36.3 | MT369932 |
| *Salmonopuntia salmiana* | 392,127 | 453 | 128,810 | 36.1 | MT369931 |
| *Tacinga palmadora* | 598,318 | 699 | 129,181 | 36.1 | MT369930 |
| *Tephrocactus weberi* | 548,088 | 632 | 128,302 | 36.1 | MT369929 |
| *Tunilla erectoclada* | 202,807 | 236 | 128,352 | 36.1 | MT369928 |

**Table S3.** Summary statistics for all chloroplast markers. Markers are ranked by phylogenetic information based on a weighed mean of relative values of number of variable sites (weight = 1), mean bootstrap (weight = 2) and distance to the full plastid tree (weight = 3).

|  | Bp | Aligned_bp | Variable | PIS | Distance | Bootstrap..Mean. | Rank |
| --- | --- | --- | --- | --- | --- | --- | --- |
| accD.cds | 1876 [1489-1927] | 1953 | 966 | 586 | 10 | 88 | 0.808798 |
| ycf1.cds | 1565 [1414-1615] | 1650 | 958 | 429 | 8 | 76 | 0.757296 |
| ndhD.cds | 1421 [1410-1421] | 1421 | 210 | 52 | 6 | 79 | 0.702738 |
| trnK-UUU.NON-CDS | 2570 [2564-2572] | 2573 | 173 | 45 | 8 | 82 | 0.676375 |
| psbE-rpl20.SPACER | 1731 [1714-1736] | 1739 | 242 | 68 | 8 | 77 | 0.663841 |
| petD.cds | 1265 [1257-1272] | 1274 | 69 | 27 | 8 | 75 | 0.645312 |
| ccsA.cds | 1008 [1007-1011] | 1011 | 110 | 49 | 8 | 73 | 0.643744 |
| clpP.cds | 359 [356-362] | 362 | 112 | 64 | 8 | 70 | 0.636547 |
| rpoC2.cds | 4101 [4101-4101] | 4101 | 165 | 47 | 8 | 69 | 0.628225 |
| rpoC1.cds | 2468 [2467-2469] | 2469 | 86 | 35 | 8 | 69 | 0.624994 |
| ycf4-cemA.SPACER | 752 [736-755] | 757 | 72 | 27 | 8 | 67 | 0.615349 |
| rps2.cds | 729 [726-729] | 729 | 77 | 24 | 10 | 71 | 0.593808 |
| rbcL-atpB.SPACER | 765 [746-768] | 768 | 122 | 27 | 12 | 79 | 0.588864 |
| rpoB.cds | 3231 [3231-3231] | 3231 | 149 | 50 | 10 | 67 | 0.585827 |
| psbB-clpP.SPACER | 547 [525-553] | 556 | 100 | 41 | 10 | 67 | 0.583404 |
| petA.cds | 963 [963-963] | 963 | 50 | 18 | 8 | 59 | 0.582963 |
| psbB.cds | 1527 [1527-1527] | 1527 | 39 | 17 | 10 | 68 | 0.580687 |
| ycf2.cds | 6065 [5933-6110] | 6145 | 1995 | 619 | 24 | 89 | 0.571429 |
| ycf3-trnS.SPACER | 804 [790-805] | 805 | 67 | 22 | 14 | 82 | 0.56304 |
| rps3.cds | 657 [657-657] | 657 | 47 | 16 | 10 | 63 | 0.561692 |
| petN-psbM.SPACER | 1135 [1130-1136] | 1137 | 79 | 23 | 12 | 71 | 0.557825 |
| matK.cds | 1530 [1524-1530] | 1530 | 104 | 27 | 12 | 68 | 0.547666 |
| ycf3.cds | 2306 [2288-2324] | 2328 | 119 | 29 | 14 | 77 | 0.546198 |
| ndhC-rbcL.SPACER | 716 [705-717] | 718 | 103 | 13 | 8 | 49 | 0.544164 |
| rpoA.cds | 1015 [1008-1020] | 1020 | 103 | 56 | 12 | 64 | 0.540493 |
| trnT-psbD.SPACER | 1022 [1008-1024] | 1025 | 57 | 16 | 10 | 57 | 0.53922 |
| ndhE-psaC.SPACER | 318 [315-328] | 328 | 70 | 26 | 14 | 75 | 0.537899 |
| ccsA-trnL.SPACER | 85 [85-85] | 85 | 20 | 7 | 10 | 57 | 0.536796 |
| trnV-UAC.NON-CDS | 394 [325-400] | 402 | 193 | 102 | 14 | 68 | 0.532145 |
| rpl32-ndhF.SPACER | 813 [775-826] | 832 | 354 | 154 | 14 | 63 | 0.52742 |
| rrn23.NON-CDS | 2838 [2834-2844] | 2844 | 89 | 29 | 12 | 62 | 0.525732 |
| psaB.cds | 2217 [2217-2217] | 2217 | 58 | 19 | 12 | 61 | 0.519294 |
| rps16.cds | 676 [672-683] | 683 | 53 | 18 | 12 | 61 | 0.519025 |
| trnF-ndhJ.SPACER | 561 [510-567] | 583 | 247 | 124 | 16 | 72 | 0.517336 |
| accD-psaI.SPACER | 599 [591-601] | 601 | 83 | 37 | 12 | 57 | 0.50916 |
| rpoB-trnC.SPACER | 1102 [1091-1108] | 1109 | 130 | 51 | 14 | 65 | 0.507178 |
| trnQ-rps16.SPACER | 650 [623-655] | 656 | 118 | 38 | 14 | 62 | 0.492441 |
| atpA.cds | 1524 [1524-1524] | 1524 | 61 | 19 | 10 | 44 | 0.491338 |
| petA-psbJ.SPACER | 1066 [1045-1072] | 1074 | 146 | 63 | 14 | 58 | 0.484191 |
| atpF-atpH.SPACER | 423 [412-425] | 425 | 41 | 15 | 12 | 51 | 0.480764 |
| trnD-trnY.SPACER | 363 [349-364] | 364 | 34 | 11 | 12 | 50 | 0.475942 |
| rpl22.cds | 543 [543-543] | 543 | 54 | 20 | 12 | 48 | 0.470875 |
| ndhH.cds | 1175 [1154-1175] | 1175 | 176 | 22 | 12 | 45 | 0.460177 |
| ycf1-rpl32.SPACER | 131 [111-137] | 146 | 124 | 78 | 14 | 50 | 0.458268 |
| rps4.cds | 606 [606-606] | 606 | 57 | 21 | 14 | 54 | 0.457901 |
| trnS-psbZ.SPACER | 365 [365-365] | 365 | 22 | 10 | 12 | 44 | 0.453201 |
| trnL-UAA.NON-CDS | 973 [952-986] | 990 | 233 | 123 | 16 | 54 | 0.449651 |
| petL-trnS.SPACER | 553 [497-556] | 563 | 197 | 56 | 16 | 58 | 0.446592 |
| rps15-ycf1.SPACER | 773 [766-774] | 775 | 296 | 129 | 18 | 61 | 0.441769 |
| atpB.cds | 1497 [1497-1497] | 1497 | 65 | 18 | 16 | 57 | 0.432615 |
| trnV-rps12.SPACER | 1352 [1339-1353] | 1353 | 173 | 16 | 12 | 38 | 0.432344 |
| petB.cds | 1370 [1368-1372] | 1372 | 44 | 12 | 14 | 47 | 0.429261 |
| cemA.cds | 690 [690-690] | 690 | 36 | 11 | 14 | 46 | 0.425246 |
| rpl33-psaJ.SPACER | 461 [450-462] | 462 | 43 | 12 | 16 | 55 | 0.423509 |
| rpl16.cds | 1465 [1450-1477] | 1480 | 107 | 31 | 18 | 62 | 0.419128 |
| infA.cds | 261 [259-264] | 264 | 57 | 27 | 16 | 52 | 0.416312 |
| atpF.cds | 1304 [1300-1310] | 1311 | 63 | 14 | 16 | 52 | 0.412812 |
| ndhF.cds | 1718 [1697-1718] | 1718 | 346 | 17 | 14 | 42 | 0.411881 |
| rps16-trnK.SPACER | 461 [456-464] | 464 | 36 | 6 | 14 | 41 | 0.405174 |
| petL.cds | 114 [113-114] | 114 | 20 | 9 | 16 | 47 | 0.392739 |
| rpl20-rps18.SPACER | 314 [290-320] | 321 | 94 | 15 | 16 | 46 | 0.390609 |
| psbD.cds | 1080 [1080-1080] | 1080 | 15 | 7 | 16 | 46 | 0.388455 |
| rpl2.cds | 828 [828-828] | 828 | 43 | 16 | 16 | 45 | 0.387133 |
| psbK-trnQ.SPACER | 338 [336-340] | 340 | 24 | 7 | 14 | 36 | 0.386716 |
| atpH-atpI.SPACER | 695 [694-695] | 695 | 25 | 8 | 14 | 34 | 0.379495 |
| psbA.cds | 1062 [1062-1062] | 1062 | 37 | 11 | 18 | 52 | 0.37629 |
| ndhC.cds | 341 [340-341] | 341 | 67 | 9 | 14 | 33 | 0.376019 |
| trnC-petN.SPACER | 560 [555-561] | 561 | 24 | 6 | 16 | 42 | 0.373205 |
| ndhE.cds | 324 [308-324] | 324 | 109 | 12 | 20 | 60 | 0.370807 |
| trnP-trnW.SPACER | 177 [176-177] | 177 | 18 | 8 | 16 | 41 | 0.369998 |
| rps11.cds | 417 [417-417] | 417 | 35 | 15 | 16 | 40 | 0.368137 |
| trnI-GAU.NON-CDS | 1022 [1021-1022] | 1022 | 23 | 7 | 18 | 50 | 0.367722 |
| trnE-trnT.SPACER | 699 [683-710] | 729 | 147 | 74 | 22 | 64 | 0.366768 |
| psaJ-trnP.SPACER | 413 [400-416] | 416 | 100 | 11 | 18 | 48 | 0.361308 |
| clpP-trnG.SPACER | 201 [200-204] | 206 | 63 | 38 | 18 | 46 | 0.361088 |
| psaI-ycf4.SPACER | 304 [303-304] | 304 | 28 | 9 | 18 | 47 | 0.357025 |
| trnK-psbA.SPACER | 228 [228-228] | 228 | 27 | 8 | 16 | 37 | 0.355017 |
| psbM-trnD.SPACER | 871 [866-877] | 877 | 43 | 13 | 18 | 45 | 0.350611 |
| trnG-UCC.NON-CDS | 756 [752-762] | 762 | 48 | 16 | 20 | 52 | 0.341922 |
| psbI-psbK.SPACER | 349 [346-351] | 351 | 54 | 19 | 20 | 51 | 0.338984 |
| rps2-rpoC2.SPACER | 237 [234-237] | 237 | 8 | 4 | 16 | 33 | 0.338958 |
| rbcL.cds | 1509 [1509-1509] | 1509 | 55 | 15 | 20 | 50 | 0.334162 |
| atpI.cds | 744 [744-744] | 744 | 21 | 6 | 16 | 31 | 0.332006 |
| psbE.cds | 309 [309-309] | 309 | 15 | 5 | 16 | 31 | 0.331737 |
| rrn16-trnV.SPACER | 226 [225-226] | 226 | 34 | 17 | 18 | 39 | 0.329216 |
| psaA.cds | 2301 [2301-2301] | 2301 | 55 | 15 | 18 | 39 | 0.328678 |
| trnF-GAA.NON-CDS | 240 [240-240] | 240 | 23 | 9 | 18 | 39 | 0.327062 |
| rps18.cds | 423 [399-425] | 425 | 82 | 33 | 22 | 56 | 0.325766 |
| cemA-petA.SPACER | 265 [265-265] | 265 | 20 | 6 | 18 | 38 | 0.322509 |
| rpl14.cds | 366 [366-366] | 366 | 41 | 10 | 18 | 37 | 0.319841 |
| rpl32.cds | 199 [199-200] | 200 | 61 | 23 | 18 | 36 | 0.319596 |
| ndhD-ccsA.SPACER | 217 [212-222] | 222 | 51 | 17 | 22 | 55 | 0.317713 |
| atpE-trnM.SPACER | 289 [282-291] | 291 | 39 | 24 | 18 | 35 | 0.31612 |
| ndhK.cds | 192 [192-192] | 192 | 22 | 5 | 18 | 36 | 0.314749 |
| atpE.cds | 402 [402-402] | 402 | 17 | 6 | 18 | 35 | 0.311273 |
| trnL-ndhG.SPACER | 230 [214-240] | 240 | 113 | 57 | 22 | 50 | 0.309756 |
| psaA-ycf3.SPACER | 471 [451-478] | 482 | 65 | 25 | 20 | 42 | 0.306892 |
| rpl4-rps8.SPACER | 164 [159-170] | 170 | 18 | 9 | 18 | 33 | 0.30459 |
| psbZ-trnG.SPACER | 365 [359-370] | 370 | 28 | 7 | 22 | 51 | 0.300039 |
| trnG-trnfM.SPACER | 231 [224-232] | 232 | 20 | 5 | 20 | 41 | 0.297761 |
| atpA-atpF.SPACER | 69 [67-69] | 69 | 11 | 7 | 18 | 31 | 0.296561 |
| psbC-trnS.SPACER | 254 [251-257] | 257 | 15 | 10 | 20 | 39 | 0.291617 |
| rpl33.cds | 204 [204-204] | 204 | 12 | 3 | 18 | 28 | 0.284248 |
| ndhA.cds | 1624 [1607-1629] | 1631 | 192 | 51 | 24 | 52 | 0.279917 |
| ndhJ-ndhK.SPACER | 139 [120-143] | 143 | 21 | 5 | 20 | 36 | 0.279035 |
| rpl20.cds | 512 [503-512] | 512 | 36 | 11 | 18 | 26 | 0.278911 |
| rps14.cds | 303 [303-303] | 303 | 16 | 9 | 24 | 54 | 0.276099 |
| rrn16.NON-CDS | 1491 [1490-1491] | 1491 | 45 | 13 | 20 | 33 | 0.269953 |
| petB-psbH.SPACER | 228 [227-228] | 228 | 18 | 5 | 18 | 24 | 0.269805 |
| trnS-psbI.SPACER | 181 [180-189] | 189 | 29 | 9 | 22 | 42 | 0.266869 |
| psbC.cds | 1422 [1422-1422] | 1422 | 36 | 7 | 20 | 32 | 0.264592 |
| rpl2-rps19.SPACER | 181 [180-182] | 182 | 25 | 6 | 20 | 32 | 0.264323 |
| rpl36-rps11.SPACER | 128 [127-130] | 130 | 8 | 2 | 18 | 22 | 0.261507 |
| trnA-UGC.NON-CDS | 887 [878-887] | 887 | 18 | 7 | 24 | 49 | 0.256834 |
| psbA-trnH.SPACER | 332 [322-338] | 343 | 98 | 44 | 24 | 45 | 0.251815 |
| trnL-trnF.SPACER | 239 [239-239] | 239 | 18 | 6 | 22 | 38 | 0.25108 |
| rps18-rpl33.SPACER | 330 [304-340] | 347 | 157 | 81 | 24 | 42 | 0.250541 |
| trnV-accD.SPACER | 148 [134-150] | 150 | 97 | 28 | 22 | 34 | 0.242023 |
| trnS-rps4.SPACER | 355 [345-355] | 355 | 25 | 6 | 22 | 34 | 0.236099 |
| ycf4.cds | 549 [549-549] | 549 | 19 | 5 | 22 | 33 | 0.232085 |
| ndhB.cds | 2165 [2141-2165] | 2165 | 252 | 72 | 24 | 37 | 0.229391 |
| ycf2-trnI.SPACER | 144 [129-146] | 147 | 58 | 17 | 22 | 31 | 0.227825 |
| psbT-psbB.SPACER | 165 [164-165] | 165 | 11 | 5 | 20 | 22 | 0.2266 |
| psaC-ndhD.SPACER | 94 [77-95] | 95 | 11 | 5 | 20 | 21 | 0.222855 |
| rps8.cds | 405 [405-405] | 405 | 23 | 6 | 22 | 30 | 0.221118 |
| atpH.cds | 265 [263-266] | 266 | 9 | 3 | 22 | 29 | 0.216565 |
| psbK.cds | 189 [189-189] | 189 | 12 | 4 | 22 | 28 | 0.213089 |
| psaC.cds | 246 [246-246] | 246 | 15 | 5 | 24 | 35 | 0.203861 |
| ndhJ.cds | 334 [327-334] | 334 | 65 | 6 | 22 | 25 | 0.202391 |
| trnI-rrn16.SPACER | 293 [287-293] | 293 | 36 | 28 | 22 | 22 | 0.197079 |
| ndhF-trnN.SPACER | 355 [339-360] | 360 | 64 | 7 | 24 | 33 | 0.196909 |
| rps15.cds | 380 [373-381] | 381 | 24 | 3 | 24 | 32 | 0.192087 |
| petG.cds | 200 [198-201] | 201 | 10 | 2 | 22 | 22 | 0.190078 |
| rps7.cds | 504 [503-504] | 504 | 38 | 9 | 24 | 31 | 0.189957 |
| trnT-trnL.SPACER | 648 [636-650] | 652 | 138 | 20 | 24 | 29 | 0.185428 |
| trnN-trnR.SPACER | 598 [598-599] | 599 | 37 | 7 | 26 | 39 | 0.183666 |
| ndhD-ndhI.SPACER | 239 [230-239] | 239 | 95 | 15 | 24 | 26 | 0.172846 |
| petG-petL.SPACER | 83 [83-83] | 83 | 19 | 4 | 24 | 25 | 0.166139 |
| rps4-trnT.SPACER | 403 [397-407] | 407 | 24 | 6 | 24 | 24 | 0.162932 |
| trnT-GGU.NON-CDS | 72 [72-74] | 74 | 5 | 2 | 22 | 14 | 0.160116 |
| psbZ.cds | 189 [189-189] | 189 | 4 | 2 | 24 | 23 | 0.158109 |
| trnS-GGA.NON-CDS | 87 [87-87] | 87 | 9 | 3 | 24 | 22 | 0.154633 |
| psbF.cds | 120 [120-120] | 120 | 4 | 1 | 22 | 12 | 0.152356 |
| trnM-trnV.SPACER | 63 [56-63] | 63 | 13 | 1 | 22 | 12 | 0.152356 |
| psaJ.cds | 129 [128-129] | 129 | 7 | 2 | 24 | 20 | 0.146873 |
| rrn5-rrn4.5.SPACER | 257 [248-257] | 257 | 14 | 2 | 24 | 20 | 0.146873 |
| rps19.cds | 150 [150-150] | 150 | 16 | 6 | 26 | 29 | 0.145944 |
| trnfM-rps14.SPACER | 152 [152-152] | 152 | 18 | 5 | 26 | 29 | 0.145675 |
| rpl16-rpl4.SPACER | 144 [144-148] | 148 | 7 | 4 | 26 | 29 | 0.145406 |
| rpoA-petD.SPACER | 147 [147-147] | 147 | 28 | 19 | 24 | 18 | 0.14396 |
| petD-petB.SPACER | 212 [210-214] | 214 | 8 | 2 | 24 | 19 | 0.143128 |
| rps14-psaB.SPACER | 125 [125-125] | 125 | 12 | 7 | 24 | 18 | 0.140729 |
| rps8-infA.SPACER | 100 [92-100] | 100 | 34 | 14 | 26 | 27 | 0.140607 |
| rpoC2-rpoC1.SPACER | 144 [144-144] | 144 | 9 | 4 | 24 | 18 | 0.139921 |
| psaI.cds | 111 [111-111] | 111 | 5 | 3 | 24 | 18 | 0.139652 |
| psbJ.cds | 123 [123-123] | 123 | 3 | 2 | 24 | 17 | 0.135637 |
| trnW-petG.SPACER | 139 [139-139] | 139 | 11 | 2 | 24 | 16 | 0.131892 |
| psbL.cds | 126 [118-127] | 127 | 10 | 1 | 24 | 16 | 0.131623 |
| ndhK-ndhC.SPACER | 53 [48-53] | 53 | 9 | 1 | 26 | 24 | 0.125871 |
| trnR-ACG.NON-CDS | 74 [73-74] | 74 | 3 | 1 | 24 | 12 | 0.116642 |
| atpI-rps2.SPACER | 189 [189-189] | 189 | 8 | 2 | 24 | 11 | 0.113166 |
| petN.cds | 153 [153-153] | 153 | 4 | 2 | 24 | 11 | 0.113166 |
| trnN-GUU.NON-CDS | 72 [72-72] | 72 | 2 | 1 | 24 | 11 | 0.112896 |
| trnG-trnR.SPACER | 140 [140-140] | 140 | 13 | 3 | 26 | 20 | 0.111428 |
| rps3-rpl16.SPACER | 229 [226-231] | 231 | 14 | 1 | 24 | 10 | 0.109151 |
| rps12.cds | 954 [954-955] | 955 | 27 | 6 | 26 | 19 | 0.108491 |
| psbN.cds | 132 [132-132] | 132 | 5 | 2 | 24 | 9 | 0.105675 |
| rrn5.NON-CDS | 121 [120-121] | 121 | 4 | 1 | 24 | 9 | 0.105406 |
| rpl23-rpl2.SPACER | 144 [140-144] | 144 | 13 | 2 | 24 | 8 | 0.10193 |
| ndhI-ndhA.SPACER | 31 [30-37] | 37 | 7 | 1 | 24 | 8 | 0.10166 |
| psbH.cds | 242 [242-242] | 242 | 11 | 1 | 24 | 8 | 0.10166 |
| ndhB-trnL.SPACER | 528 [512-530] | 530 | 120 | 20 | 28 | 23 | 0.091527 |
| ndhG.cds | 380 [380-380] | 380 | 48 | 6 | 26 | 13 | 0.086019 |
| psbN-psbT.SPACER | 60 [60-60] | 60 | 3 | 1 | 26 | 13 | 0.084673 |
| rpoC1-rpoB.SPACER | 26 [26-26] | 26 | 9 | 3 | 28 | 22 | 0.083205 |
| trnR-atpA.SPACER | 101 [100-101] | 101 | 29 | 1 | 26 | 11 | 0.077182 |
| ndhA-ndhH.SPACER | 81 [81-81] | 81 | 4 | 4 | 26 | 9 | 0.070499 |
| rpl23.cds | 191 [189-191] | 191 | 18 | 2 | 26 | 9 | 0.069961 |
| psbH-psbN.SPACER | 86 [86-86] | 86 | 10 | 1 | 26 | 9 | 0.069691 |
| ndhH-rps15.SPACER | 102 [90-102] | 102 | 12 | 1 | 26 | 8 | 0.065946 |
| psbI.cds | 111 [111-111] | 111 | 3 | 1 | 26 | 8 | 0.065946 |
| trnR-rrn5.SPACER | 256 [255-257] | 257 | 16 | 1 | 28 | 15 | 0.056449 |
| trnL-ycf2.SPACER | 142 [136-142] | 142 | 42 | 7 | 28 | 14 | 0.054319 |
| rpl36.cds | 123 [123-123] | 123 | 10 | 1 | 28 | 12 | 0.045213 |
| trnC-GCA.NON-CDS | 71 [71-71] | 71 | 4 | 1 | 28 | 12 | 0.045213 |
| trnI-rpl23.SPACER | 106 [105-106] | 106 | 11 | 2 | 26 | 2 | 0.043743 |
| trnY-GUA.NON-CDS | 123 [123-123] | 123 | 6 | 1 | 26 | 2 | 0.043474 |
| psbJ-psbL.SPACER | 103 [103-103] | 103 | 5 | 0 | 26 | 1 | 0.03946 |
| rrn23-trnA.SPACER | 149 [146-149] | 149 | 5 | 0 | 26 | 1 | 0.03946 |
| rps11-rpoA.SPACER | 71 [71-71] | 71 | 9 | 2 | 28 | 10 | 0.037992 |
| trnT-UGU.NON-CDS | 73 [73-73] | 73 | 4 | 1 | 26 | 0 | 0.035984 |
| psbT.cds | 108 [108-108] | 108 | 4 | 1 | 28 | 9 | 0.033977 |
| ndhI.cds | 271 [271-271] | 271 | 24 | 1 | 28 | 8 | 0.030232 |
| rps7-ndhB.SPACER | 228 [224-228] | 228 | 38 | 0 | 28 | 2 | 0.007491 |
| rrn4.5-rrn23.SPACER | 99 [98-99] | 99 | 5 | 0 | 28 | 1 | 0.003745 |
| trnL-UAG.NON-CDS | 80 [80-80] | 80 | 1 | 0 | 28 | 1 | 0.003745 |
| trnM-CAU.NON-CDS | 150 [150-150] | 150 | 7 | 0 | 28 | 1 | 0.003745 |
| trnV-GAC.NON-CDS | 72 [72-72] | 72 | 2 | 0 | 28 | 1 | 0.003745 |
| trnY-trnE.SPACER | 52 [52-52] | 52 | 2 | 0 | 28 | 1 | 0.003745 |
| psbM.cds | 105 [105-105] | 105 | 1 | 0 | 28 | 0 | 0 |
| rrn4.5.NON-CDS | 103 [99-103] | 103 | 4 | 0 | 28 | 0 | 0 |

**Table S4.** Summary statistics for the top 10 markers with suitable size for PCR amplification. Markers are ranked by phylogenetic information based on a weighed mean of relative values of number of variable sites (weight = 1), mean bootstrap (weight = 2) and distance to the full plastid tree (weight = 3).

|  | Bp | Aligned_bp | Variable | PIS | Distance | Bootstrap..Mean. |
| --- | --- | --- | --- | --- | --- | --- |
| clpP.cds | 359 [356-362] | 362 | 112 | 64 | 8 | 70 |
| ycf4-cemA.SPACER | 752 [736-755] | 757 | 72 | 27 | 8 | 67 |
| rps2.cds | 729 [726-729] | 729 | 77 | 24 | 10 | 71 |
| rbcL-atpB.SPACER | 765 [746-768] | 768 | 122 | 27 | 12 | 79 |
| psbB-clpP.SPACER | 547 [525-553] | 556 | 100 | 41 | 10 | 67 |
| petA.cds | 963 [963-963] | 963 | 50 | 18 | 8 | 59 |
| ycf3-trnS.SPACER | 804 [790-805] | 805 | 67 | 22 | 14 | 82 |
| rps3.cds | 657 [657-657] | 657 | 47 | 16 | 10 | 63 |
| ndhC-rbcL.SPACER | 716 [705-717] | 718 | 103 | 13 | 8 | 49 |
| ndhE-psaC.SPACER | 318 [315-328] | 328 | 70 | 26 | 14 | 75 |
